# Quantification of Thermal Oxidation in Metallic Glass Powder using Ultra-small Angle X-ray Scattering

**DOI:** 10.1038/s41598-019-43317-0

**Published:** 2019-05-02

**Authors:** Tanaji Paul, Linqi Zhang, Sourabh Biswas, Archana Loganathan, Matthew G. Frith, Jan Ilavsky, Ivan Kuzmenko, Jim Puckette, A. Kaan Kalkan, Arvind Agarwal, Sandip P. Harimkar

**Affiliations:** 1 School of Mechanical and Aerospace Engineering, Oklahoma State University, Stillwater, OK 74078 United States; 2 Plasma Forming Laboratory, Department of Mechanical and Materials Engineering, Florida International University, Miami, FL 33174 United States; 3 X-ray Science Division, Advanced Photon Source, Argonne National Laboratory, 9700 South Cass Avenue, Argonne, IL 60439 United States; 4 Boone Pickens School of Geology, Oklahoma State University, Stillwater, OK 74078 United States

**Keywords:** Mechanical engineering, Synthesis and processing, Metals and alloys

## Abstract

In this paper, the composition, structure, morphology and kinetics of evolution during isothermal oxidation of Fe_48_Cr_15_Mo_14_Y_2_C_15_B_6_ metallic glass powder in the supercooled region are investigated by an integrated *ex*-*situ* and *in*-*situ* characterization and modelling approach. Raman and X-ray diffraction spectra established that oxidation yielded a hierarchical structure across decreasing length scales. At larger scale, Fe_2_O_3_ grows as a uniform shell over the powder core. This shell, at smaller scale, consists of multiple grains. Ultra-small angle X-ray scattering intensity acquired during isothermal oxidation of the powder over a wide Q-range delineated direct quantification of oxidation behavior. The hierarchical structure was employed to construct a scattering model that was fitted to the measured intensity distributions to estimate the thickness of the oxide shell. The relative gain in mass during oxidation, computed theoretically from this model, relatively underestimated that measured in practice by a thermogravimetric analyzer due to the distribution in sizes of the particles. Overall, this paper presents the first direct quantification of oxidation in metallic glass powder by ultra-small angle X-ray scattering. It establishes novel experimental environments that can potentially unfold new paradigms of research into a wide spectrum of interfacial reactions in powder materials at elevated temperatures.

## Introduction

Progress of human civilization has been inextricably associated with the discovery of novel materials that exhibit exceptional properties. One of the recent examples of such a class of materials is metallic glasses^1–3^. These are solid metallic alloys that exhibit an atomic structure that is fundamentally different from polycrystalline materials^4,5^. Metallic glasses possess a homogeneous disordered atomic structure that is devoid of conventional crystalline defects such as dislocations and grain boundaries^6,7^. As a result of this defect-free, disordered atomic structure, metallic glasses manifest mechanical^8,9^, tribological^10,11^ and magnetic properties^12,13^ that are superior to those of their polycrystalline counterparts. Among them, iron-based metallic glasses are particularly attractive^14,15^, being comparatively inexpensive in materials costs as compared to palladium and zirconium-based ones. For example, Fe_48_Cr_15_Mo_14_Y_2_C_15_B_6_ metallic glass exhibits exceptionally high elastic modulus of 200 GPa and compressive fracture strength of 3200 MPa. The hardness of this metallic glass is also high (13 GPa)^16–19^. Metallic glasses in the Fe-Si-B alloy system also possess excellent soft magnetic properties such as high permeability and low core loss. The saturation induction and maximum dc permeability of *Metglas*^R^ 2605SA1 are 1.56T and 600 kNA^−2^ respectively^20–22^. These outstanding properties of metallic glasses harbor the potential to be utilized in a wide spectrum of applications spanning hard dies and tools, wear resistant cutting materials and transformer cores thereby ushering rapid technological and industrial advancement.

In order that an extensive commercial utilization of metallic glasses be viable, it is critical to evaluate these materials for their environmental stability at elevated temperatures^23^. It is imperative that metallic glasses exhibit adequate resistance against detrimental oxygen environments to be able to execute satisfactory performance^24^. For example, it is necessary to anneal metallic glass ribbons under a magnetic field to reduce stress and attain the requisite domain structure^25^. In most industrial manufacturing processes an ideal vacuum annealing environment is seldom achievable^26^. The result is an occurrence of oxidation on the surface of metallic glasses that introduces magnetic anisotropy and pinning centers that severely deteriorates soft magnetic properties^26^. This phenomenon of oxidation in metallic glasses is fundamentally different than that in polycrystalline materials and is expected to be more uniform due to the absence of grain boundaries^23^. Additionally, it has been observed that the composition of oxide films naturally formed in air on metallic glasses exerts strong influence on their passivity and corrosion behavior^27,28^. From a different perspective, thermally induced oxidation is a major chemical approach to synthesize oxide thin films for applications such as sensors, catalysts, insulators and bioimplants^29^. Oxidation of metallic glasses have also been reported to yield a ten times improvement in the wear resistance of these materials^30–32^. This could be utilized by metallic glass alloys and coatings fabricated by a number of processing techniques such as laser cladding and spark plasma sintering^33–35^. Thus, the successful manufacturing and applications of metallic glasses mandates a fundamental understanding of their behavior in oxygen-containing atmospheres during thermally activated processes.

The utilization of metallic glass alloy systems during manufacturing as well as applications is most extensive in the temperature regime of the supercooled liquid region (SLR) or, in other words, between the glass transition temperature T_g_ and the onset crystallization temperature T_x_^36^. This temperature regime enables harnessing the drastic decrease in viscosity of metallic glasses^37^. Indeed these materials have been observed to exhibit viscosities varying from 1 × 10^12^ Pas at T_g_ to 1 × 10^5^ Pas at T_x_^38^. Superplastic forming of metallic glasses can thus be achieved to fabricate complex near net components^39^. Additionally, this temperature regime, which is an evidence of the remarkable stability of the metallic glass against crystallization, restricts devitrification and permits preservation of the amorphous structure of the material and hence its superior properties discussed earlier^40^. Thus investigation of the response of metallic glasses to oxidation in the supercooled liquid region without devitrification of its structure is of paramount importance.

An accurate investigation of the oxidation behavior of metallic glasses in the supercooled liquid region warrants the selection of materials and employment of characterization techniques that enable precise and reliable identification of chemistry and quantification of the volume of oxide species evolved. It is established that oxidation is a slow, diffusion based phenomenon that occurs on the surface of materials. Reported examinations of oxidation are mostly limited to bulk and ribbon forms of metallic glasses^41–43^. The relatively lower specific surface area of these materials result in the evolution of a minute volumes of oxides^44^. Consequently, reliable quantification of oxidation is significantly impeded. It is thus commonplace to observe that such quantification is generally performed by indirect means such as estimation of weight^45^. This impediment is especially severe during the initial stages of oxidation, which, in fact, provide deeper insight into the reaction and hence arouses further intrigue. Conventional characterization techniques too induce drawbacks in analyzing the oxidation behavior of these materials. Scanning electron microscopy (SEM), for example, can resolve the morphology of films evolved only over a prolonged duration of oxidation^44^. Laboratory scale desktop X-ray diffraction (XRD) instruments while capable of determining the structure of oxides, the intensity of scattering is not representative of the minute volume of oxides formed^23^.

Circumvention of these problems validates both the utilization of metallic glass in powder form that exhibits higher specific surface area as well as characterization techniques that can directly enumerate the evolution of oxides, particularly at the initial stages. In this regard, synchrotron-based *in*-*situ* X-ray scattering techniques are particularly attractive^46,47^. For example, ultra-small angle X-ray scattering (USAXS) is a powerful technique that enables the acquisition of a high intensity of scattering^48,49^ even from a minute volume of structural features in metallic glasses^50^. Recent development of experimental environments have equipped the scientific community to acquire USAXS intensities *in*-*situ* during thermally-activated phenomena^51^. The USAXS intensity, I(Q) can be acquired over a broad range of the scattering vector, Q^52^. The dimensions of the scattering features are directly correlated to the scattering vector as 2*π*/Q^53^. Thus the distribution of USAXS intensity, I(Q) over the scattering vector, Q enables analysis of hierarchical structural features across a wide span of length scales from hundreds of micrometres to a few nanometres. The nature of the USAXS intensity distribution as a function of the scattering vector is also characteristic of the morphology of the scattering features due to the unique form factor each generates. For example, each of spheres, disks and cylinders of the same material will generate characteristic form factors and hence intensity distributions that can be utilized to distinguish between features of different morphologies^54^. The measured intensity distributions can be further fitted by small angle scattering analysis software tools based upon physical models that replicate the evolution of reactions^55,56^. This in turn can be utilized to yield quantitative information on the evolution of the scattering features. In summary, while *ex*-*situ* characterization techniques are suitable for the identification of chemistry and structure of oxides, *in*-*situ* techniques can enable quantification of time dependent evolution. These strongly complement each other and, utilized in conjunction, provide an excellent procedure of accurately establishing the oxidation behavior of metallic glass powder throughout the initial stages towards conclusion.

The present paper aims to establish a fundamental understanding of the oxidation behavior of metallic glass powder by investigating the composition, structure, morphology and isothermal evolution by an integrated approach employing complementary *ex*-*situ* and *in*-*situ* characterization techniques. The chemistry of the oxide species and their morphology are presented followed by an analysis of their microstructure. These are employed to construct a physical model to interpret the distribution in intensity of ultra-small angle X-rays scattered *in*-*situ* during isothermal evolution. The theoretical computation from this distribution is compared with thermogravimetric measurement to establish the isothermal oxidation behavior of the metallic glass powder. This paper, to the best of the authors’ knowledge, presents the first report of oxidation kinetics of metallic glass powder by ultra-small angle X-rays scattering, complemented by Raman spectroscopy and X-ray diffraction techniques. En route, novel experimental environments and analytical methods are established. In principle, these harbor the potential to be utilized for investigating the mechanism and kinetics of a wide spectrum of slow, surface controlled reactions beyond oxidation, in powder materials not limited to metallic glasses. Indeed, the presented framework manifests the prospect to unfold new paradigms of surface research on heterogeneous catalysis, adsorption, adhesion and so forth at ambient and elevated temperatures based upon combined *ex*-*situ* and *in*-*operando* characterization capabilities.

## Results and Discussion

### Powder morphology and structure

The morphology of the pristine Fe_48_Cr_15_Mo_14_Y_2_C_15_B_6_ metallic glass powder consists of particles of various shapes with the majority of them being spherical. The sizes of the particles measured from several SEM micrographs (see Supplementary Fig. S1) showed that more than 90% of the particles exhibit a diameter ranging from 20 μm to 60 μm with the mean diameter, D being 40 μm^57^.

This paper is aimed at investigating the oxidation behavior of this metallic glass powder within the supercooled liquid region of the material, without the occurrence of crystallization. Thus, in order to determine the temperatures of isothermal time dependent oxidation experiments, the thermal behavior of the material was examined first. The isochronal DSC trace of the metallic glass powder, measured at a heating rate of 50 °C min^−1^ (see Supplementary Fig. S2a) exhibited a glass transition temperature T_g_ of about 570 °C and a crystallization onset temperature T_x_ of about 660 °C. Based on this thermogram, the temperatures of isothermal oxidation were determined to be 580 °C and 650 °C, completely within the glass transition and onset crystallization temperatures. Two more isochronal annealing experiments were conducted at the same heating rate, with the powder being heated up to 580 °C and 650 °C. The XRD spectra acquired from the powder in both pristine and annealed conditions (see Supplementary Fig. S2b) exhibited absence of crystallization as confirmed by the diffused peak in each spectra, characteristic of fully amorphous materials.

### Oxide chemistry

**Figure 1:**
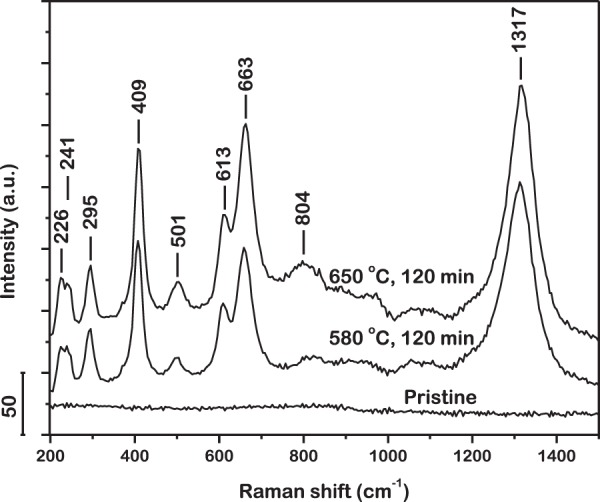
Representative Raman spectra acquired from surface of pristine and oxidized metallic glass powder particles. Surface of pristine powder particles is clean, devoid of detectable oxides. Oxidation at 580 °C and 650 °C results primarily in formation of Fe_2_O_3_ which grows as a uniform shell over the surface of the particles.

The iron-rich section of the iron-oxygen phase diagram^58^ (see Supplementary Fig. S3) shows that at the temperatures of isothermal oxidation experiments employed in this investigation, 580 °C and 650 °C, the oxides that are thermodynamically stable are FeO, Fe_2_O_3_ and Fe_3_O_4_. It is thus expected that the oxidation of this metallic glass powder would yield oxides among the aforementioned ones. The surface of the pristine and oxidized metallic glass powder particles was examined by Raman spectroscopy. Spectra were acquired from multiple spots on individual particles among which, one, representative of the entire powder, is presented for each sample in Fig. 1. No Raman features are observed in the spectrum acquired from the pristine powder. This confirms that the surface of the pristine metallic glass powder is clean, devoid of any detectable oxides or other compounds on the metal surface. The spectra acquired from the surface of the powder oxidized at 580 °C and 650 °C for 120 min, each exhibit peaks that have been identified by their frequencies. The peaks at 226, 241, 295, 409, 501, 613 and 1317 cm^−1^ all correspond to Fe_2_O_3_ oxide^59,60^. Only the peak at 663 cm^−1^ is assigned to Fe_3_O_4_ oxide^61^. A low intensity peak at 804 cm^−1^ is observed only for the sample oxidized at 650 °C for 120 min which is attributed to the formation of B_2_O_3_. This observation agrees with the literature that boron is oxidized at a temperature higher than 600 °C^62^. B_2_O_3_ has also been observed upon oxidation of Fe-based metallic glasses containing boron^43,44,63,64^. Thus it can be concluded that the oxide phases grow as a shell over the entire surface of the metallic glass powder particles. This is reasonable as the powder possesses a homogeneous amorphous structure and all sites on the surface are equally preferred for the growth of oxides. Additionally, according to the Raman spectra (Fig. 1), the major constituent of the oxide shell is Fe_2_O_3_.

**Figure 2:**
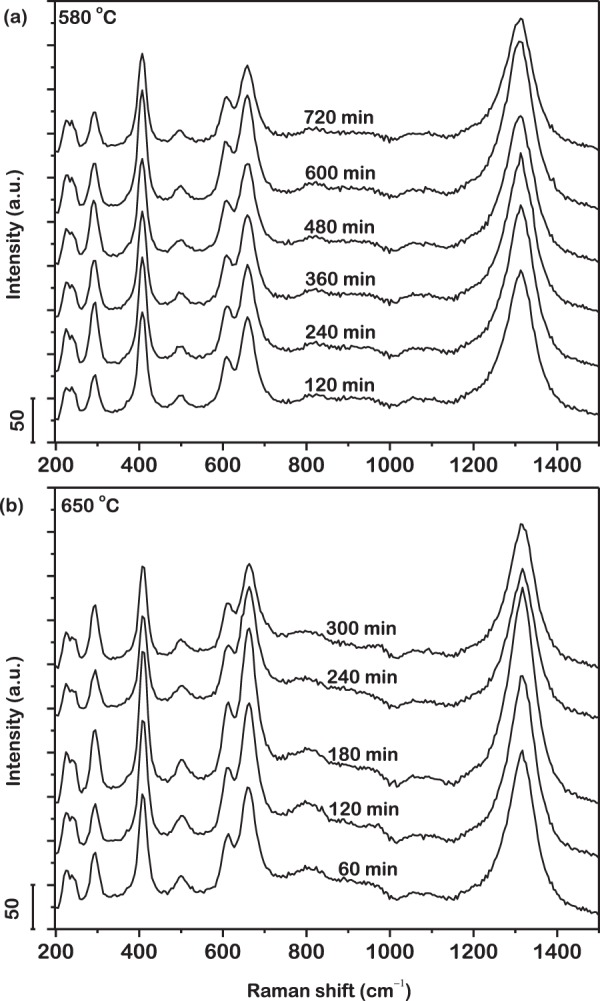
Raman spectra acquired from the surface of the metallic glass powder isothermally oxidized at (**a**) 580 °C for 720 min and (**b**) 650 °C for 300 min. Oxide grows as a uniform shell consistently over the surface of the powder particles. Irregularity in intensity of peaks with increasing time of isothermal oxidation is a possible outcome of optical interference due to multiple reflections of incident laser in oxide shell.

Raman spectra acquired from the surface of the metallic glass powder isothermally oxidized for systematically increasing duration of time at 580 °C for 120; 240; 360; 480; 600 and 720 min and at 650 °C for 60; 120; 180; 240 and 300 min are presented in Fig. 2. The oxides are consistently observed to grow during the entire duration of isothermal oxidation at both temperatures. No noticeable peak shift is detected which suggests the uniformity of oxide growth with time, over the entire surface of the metallic glass powder particles.

With increasing time of isothermal oxidation, the volume of oxide shell growing on the surface of the powder is expected to increase, thereby resulting in an increase in intensity of the characteristic peaks in the Raman spectra. However, as presented in Fig. 2, the trend in intensity of the peaks are observed to be irregular at both temperatures. This irregularity in intensity of the peaks can possibly be an outcome of optical interference caused by multiple reflections of the incident laser in the oxide shell. The constructive or destructive interference, based on the thickness of the oxide shell regulates the intensity of the Raman-scattered radiation emitted to the far field. Indeed, oscillation of the intensities of the peaks in the Raman spectrum with thickness of oxide has been reported previously^65,66^. Variation in the particle color was also observed, that is suggestive of the same.

**Figure 3:**
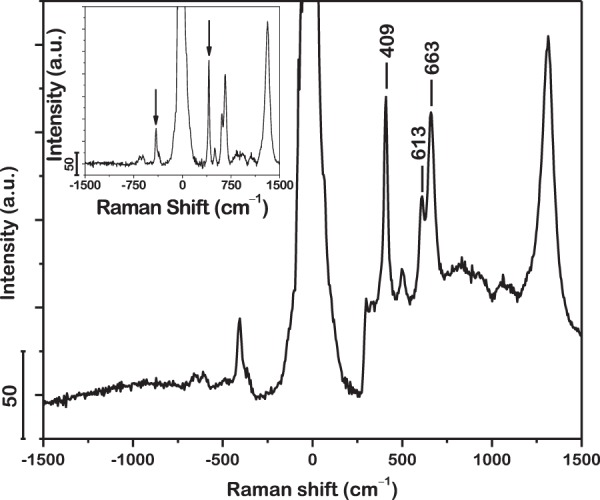
Estimation of laser-induced rise in temperature of sample from anti-Stokes-to-Stokes intensity ratio of peak at 409 cm^−1^ in Raman spectrum acquired from surface of powder oxidized at 580 °C for 720 min. *Inset* represents same spectrum after background subtraction. Temperature rise with incident laser power of 3 mW is computed to be 278 °C, too low to induce oxidation.

Heating of sample due to interaction with laser is frequently encountered in Raman spectroscopy^67^. It may result in artifacts, such as oxidation of sample and temperature-induced peak shifts. In the present investigation, the absence of Raman peaks in the spectrum acquired from the surface of the pristine metallic glass powder particles suggests that no oxides are formed during excitation where the laser power is optimized to 3 mW for highest signal counts without artifacts. On the other hand, oxidation of the powder was clearly observed when the laser power was increased to 4 mW (not shown here). Hence the presence of laser heating during the acquisition of Raman spectra was anticipated, although it was not high enough to drive oxidation. To this end, the laser-induced rise in temperature of the sample was estimated from the anti-Stokes-to-Stokes intensity ratio, I_aS_/I_S_, which is approximately the Boltzmann factor^68,69^. Figure 3 presents the Raman spectrum acquired from the surface of the powder oxidized at 580 °C for 720 min with an incident laser power of 3 mW. The most intense anti-Stokes peak, being at −409 cm^−1^, was used for calculation of temperature. I_aS_/I_S_ is found to be 0.342 after background subtraction (inset, Fig. 3). Hence, the temperature is estimated to be 278 °C, which is too low to induce oxidation. Similarly, the temperature is estimated to be 152 °C for an incident laser power of 1 mW where the corresponding Raman mode was observed at 410.5 cm^−1^. Accordingly, by linear extrapolation, the peak is estimated to be at 412 cm^−1^ at room temperature. Therefore, the acquisitions of Raman spectra in this investigation were not subject to significant spectral shifts due to laser heating and the assignments of the peaks are thus accurate.

### Oxide structure

**Figure 4:**
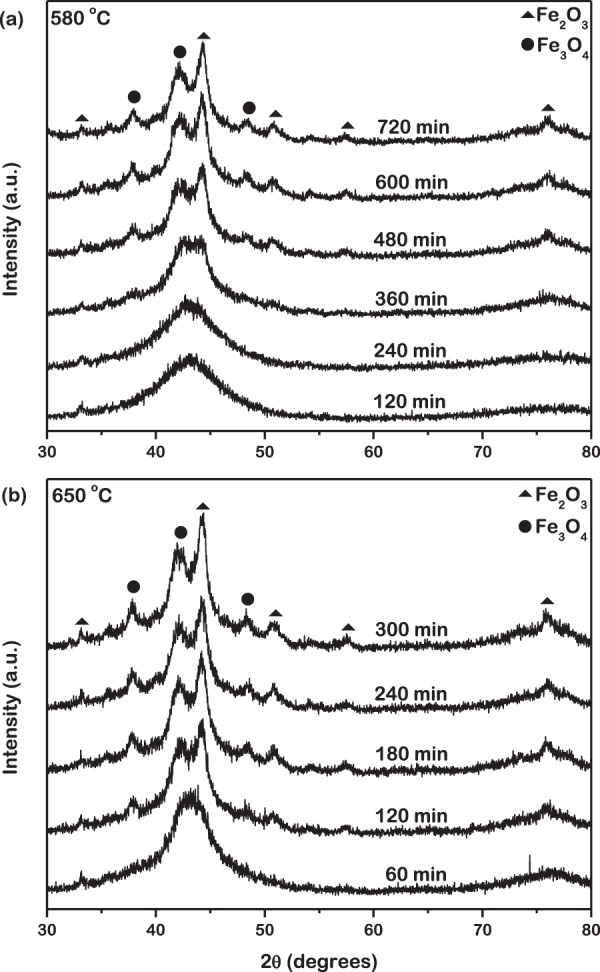
XRD spectra acquired from metallic glass powder isothermally oxidized at (**a**) 580 °C for 720 min and (**b**) 650 °C for 300 min. Oxide shell is confirmed to be polycrystalline, consisting of multiple grains.

In order to further analyze the oxidation behavior of the metallic glass powder particles, the structure of the oxides was investigated by X-ray diffraction. The XRD spectra acquired from the metallic glass powder isothermally oxidized at 580 °C for 720 min and at 650 °C for 300 min are presented in Fig. 4. In addition to the diffused peak as in the pristine powder, these spectra exhibit sharper peaks, superimposed on the amorphous background. These peaks are identified to be Fe_2_O_3_ and Fe_3_O_4_ oxides. No peaks corresponding to B_2_O_3_ were observed in the XRD spectra owing to the minor amount formed as exhibited by the Raman spectra. The XRD spectra thus confirm the results obtained from Raman spectroscopy. During isothermal oxidation at the lower temperature of 580 °C for shorter duration of time up to 240 min the volume of the oxide shell is possibly too low to be detectable in the XRD spectra. This is also observed during isothermal oxidation at 650 °C for the shortest duration of time of 60 min. With an increase in duration of time of isothermal oxidation at both temperatures, the intensity of the characteristic peaks gradually increase, that suggests an increase in the volume of the oxide shell growing on the surface of the powder.

Hence from the Raman and XRD spectra it can be concluded that the growth of oxides on the surface of the metallic glass powder has a hierarchical structure. First, the growth occurs as a shell over the entire surface of the powder. Second, this oxide shell is polycrystalline, or in other words, constitutes of multiple oxide grains. However, both Raman and XRD spectra are incapable of providing quantitative information on the trend in volume of oxides formed as a function of time during isothermal oxidation.

### USAXS interpretation of hierarchical oxide structure

In order to enumerate the isothermal evolution of oxide in this metallic glass powder, *in*-*situ* ultra-small angle X-ray scattering (USAXS) was employed. The number of particles subjected to exposure in the beam is of the order of thousand and hence is representative of the entire sample of powder. This powder consists of non-interacting, mostly spherical particles with an average diameter, D of about 40 μm which yields D^−1^ of 0.0000025 Å^−1^. The USAXS intensity, I(Q) acquired from this pristine metallic glass powder at ambient temperature over a scattering vector, Q-range of 0.0005 Å^−1^ to 0.2 Å^−1^ (see Supplementary Fig. S4), in accord with the expected Q-dependence for a dilute solution of identical uniform spheres at Q ≫ D^−1^, can be observed to decay following a power-law as Q^−4^ ^70^. This confirms that the powder consists of particles with a smooth surface devoid of any structural features as shown by the Raman and XRD spectra. Over the Q-range above 0.1 Å^−1^, a constant, low-intensity background arising from the detector and environment is observed, which is independent of Q and not related to the morphology and structure of the powder particles and the oxides formed on their surface.

**Figure 5:**
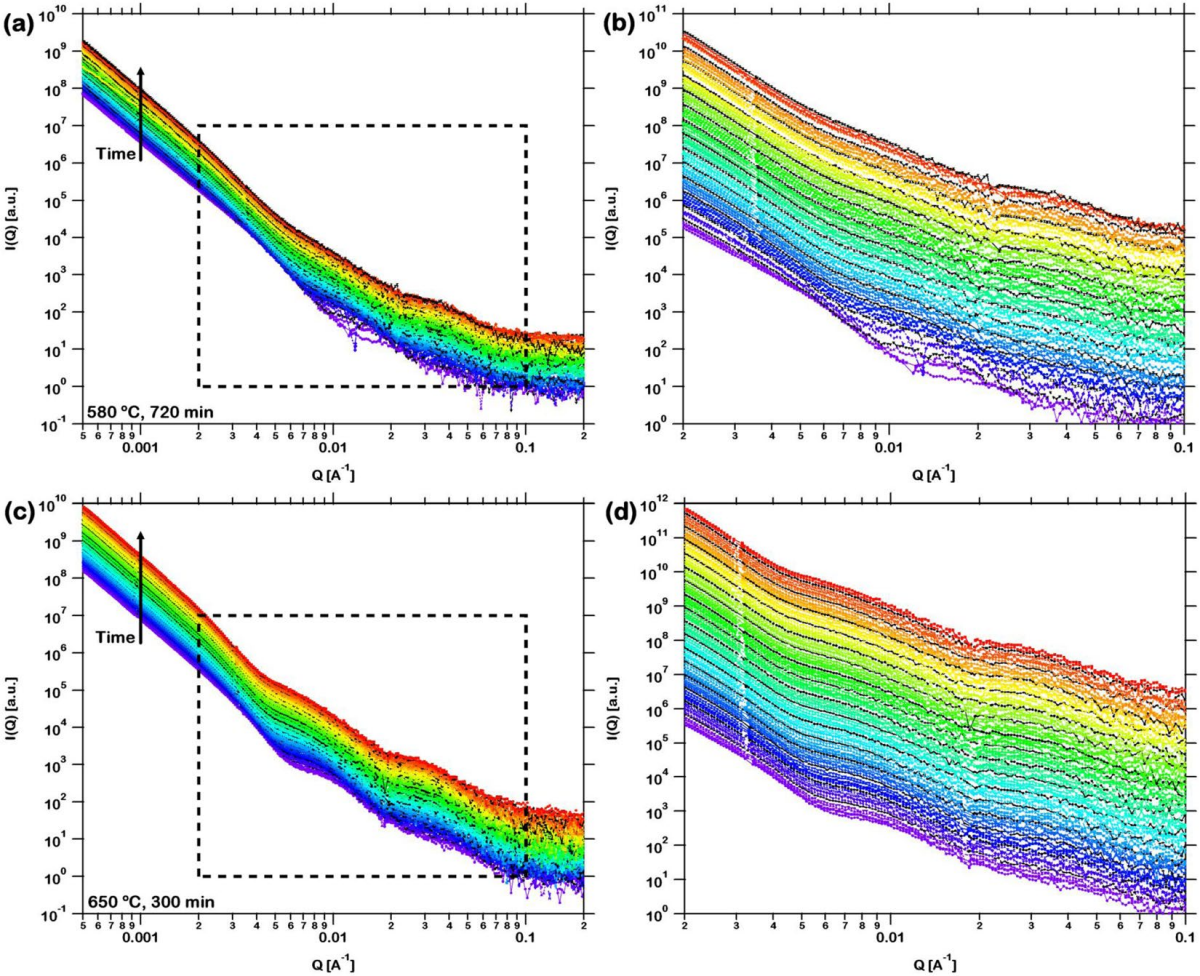
USAXS intensity distributions acquired *in*-*situ* from powder during isothermal oxidation at (**a**) 580 °C for 720 min and (**c**) 650 °C for 300 min. Duration between two consecutive measurements is approximately 3 min. In order to separate the distributions for visual clarity, all the acquired intensities are multiplied by a factor of 1.2 and only one among every three consecutive measurements are presented. Further, over the Q-range representative of the evolution of oxides, intensity distributions acquired at (**b**) 580 °C for 720 min and (**d**) 650 °C for 300 min (enclosed in black *dashed square*) are magnified. Every fifteenth distribution acquired at both temperatures is represented in black.

The evolution of oxides during *in*-*situ* isothermal time dependent experiments is manifested by the USAXS intensity distributions acquired at 580 °C for 720 min and 650 °C for 300 min as presented in Fig. 5. These intensities are calibrated on relative scale as the motivation of the present investigation is to enumerate the average volume of oxide shell formed on the particles^71^. Based on the intensity, I(Q) measured as a function of the scattering vector, Q, the characteristics of scattering, corresponding interpretation as representative of the hierarchical structure of the oxide over decreasing length scales and their evolution with increase in time during isothermal oxidation are discussed across four discrete Q-ranges as follows.

First, over the Q-range from 0.0005 Å^−1^ to 0.002 Å^−1^, scattering results from the oxidized particles. The average diameter of the particles, 40 μm is at a length scale larger than that resolved in the present USAXS measurements. It is clearly observed that with increase in time of *in*-*situ* isothermal experiments, the size of the particles themselves do not change appreciably, as evidenced by the intensity distributions at both temperatures. As a result, I(Q) continues to decay following the power-law as Q^−4^, identical to that presented for the particles at room temperature (see Supplementary Fig. S4).

Second, over the Q-range from 0.002 Å^−1^ to 0.02 Å^−1^, scattering results from the Fe_2_O_3_ oxide that grows as a uniform shell over the entire surface of the metallic glass powder particles. The length scale of the oxide shell can be estimated from this Q-range to be approximately between few tens to few hundreds of nanometres. As a result, the oxidized metallic glass powder exhibits a core-shell structure with the powder particle behaving as the core and the oxide behaving as the shell. A form factor is produced due to the difference in X-ray scattering contrasts of the core and the shell. In such a form factor the characteristic Fourier-Bessel oscillations^72^, as observed in the intensity distributions, convey quantitative information on the thickness of the oxide shell. During *in*-*situ* isothermal oxidation at both temperatures, with increase in time these oscillations shift towards lower Q, suggesting a corresponding increase in thickness of the oxide shell.

Third, over the Q-range from 0.02 Å^−1^ to 0.1 Å^−1^, scattering occurs due to the multiple grains in the polycrystalline oxide shell. The length scale of the oxide grains can be computed from this Q-range to be approximately between few to few tens of nanometres. The intensity distributions in this Q-range exhibit the well-recognized Guinier ‘knee’ that is characteristic of the radius of gyration of the grains^73^ followed by the Porod power-law slope. With increase in time during isothermal oxidation, small nuclei of oxides are formed while pre-existing grains grow into larger ones. Thus the evolution of sizes of the grains has an irregular trend and consequently the variation in intensity distribution is less conspicuous.

Finally, over the Q-range from 0.1 Å^−1^ to 0.2 Å^−1^, a constant background scattering is manifested. This originates from the instrument and environment, without any dependence on Q. This is confirmed by the fact that the measured intensities are significantly lower at this Q-range and does not evolve with time of isothermal oxidation at both temperatures.

In summary, the evolution of the hierarchical structure of the metallic glass powder with time during isothermal oxidation at both 580 °C and 650 °C across decreasing length scales is based on a powder core over which an oxide grows as a uniform shell which further consists of multiple grains. This hierarchical structure is utilized to construct a physical model under the aegis of which the fitting of parameters enables the quantitative analysis of oxidation in this material as described in the following section.

### USAXS quantitative modelling

**Figure 6:**
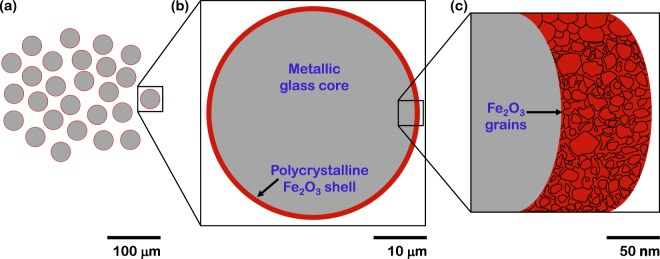
Schematic physical model representing hierarchical structure over decreasing length scales used for USAXS quantitative modelling. It consists of cross sections of (**a**) ensemble of non-interacting oxidized particles, (**b**) individual particles with powder core and oxide shell, and (**c**) multiple grains within the oxide shell.

The physical model representing the hierarchical structure is depicted by a schematic in Fig. 6. At large length scale (small Q), scattering is representative of the ensemble of a large number of non-interacting oxidized particles (Fig. 6a). With further decrease in length scale (medium Q) individual oxidized particles with distinct powder core and oxide shell can be distinguished (Fig. 6b). A section is made to clarify the internal structure. At small length scale (large Q), the scatterers are the multiple grains in the oxide shell (Fig. 6c).

**Figure 7:**
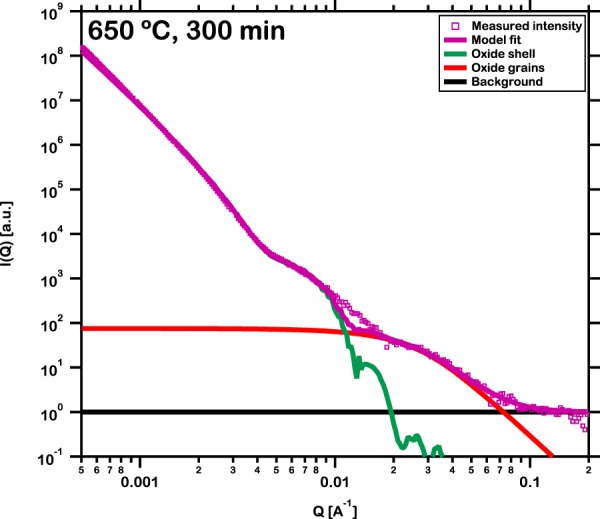
Illustration of representative fitting routine performed on the USAXS intensity acquired from the powder oxidized at 650 °C for 300 min utilizing the three components of the scattering model, the oxide shell, grains in the shell and a constant background. Small misfit results from the distribution in the diameter of the particles and hence of the thickness of the oxide shell.

In order to achieve consistency with the physical characteristics three components are utilized to construct the scattering model. In the first component, the mean diameter was fixed at 40 μm. The standard deviation was obtained from fitting the scattering intensity acquired from the pristine powder at room temperature. This was kept constant thereafter. The X-ray scattering length densities of the Fe_48_Cr_15_Mo_14_Y_2_C_15_B_6_ core and Fe_2_O_3_ shell were computed to be 60.24 × 10^10^ cm^−2^ and 42.32 × 10^10^ cm^−2^ respectively while that of air as solvent was kept zero. It is to be noted here that while the major constituent of the oxide shell is Fe_2_O_3_, both Raman and XRD spectra detected the presence of Fe_3_O_4_ as well. However, the X-ray scattering length density of Fe_3_O_4_, 41.68 × 10^10^ cm^−2^, is almost equal to that of Fe_2_O_3_. Hence, in relevance to USAXS, these oxides are similar and thus the thickness estimated from the intensity distributions are also accurate. In the second component, the Porod power-law slope was fixed at −4 and the radius of gyration was fitted. The third component accounted for the background which was set to a constant value at the beginning of the fitting routine. Figure 7 presents the result of a representative fitting routine performed on the USAXS intensity acquired from the metallic glass powder oxidized at 650 °C for 300 min. The model can be observed to be in good agreement with the measured USAXS intensity. The small misfit results from the fact that the present model is utilized to estimate a single value of the thickness of the oxide shell growing over all the particles. In practice, however, in a distribution of diameters of the powder cores with a mean and standard deviation, the thickness of the oxide shell is not constant and is dependent on the diameter of the individual particles. Thus, although the model underestimates the thickness of the oxide shell and hence the USAXS intensity near the Q-range of the misfit, the overall concurrence yields a good estimate of the oxide shell thickness. This fitting routine is employed to all the USAXS intensity distributions measured during isothermal oxidation at 580 °C for 720 min and 650 °C for 300 min to estimate the evolution of thickness of oxide shell (see Supplementary Fig. S5) and validate the mass gain by thermogravimetric analysis as described in the following section.

### Quantitative validation of oxide evolution

**Figure 8:**
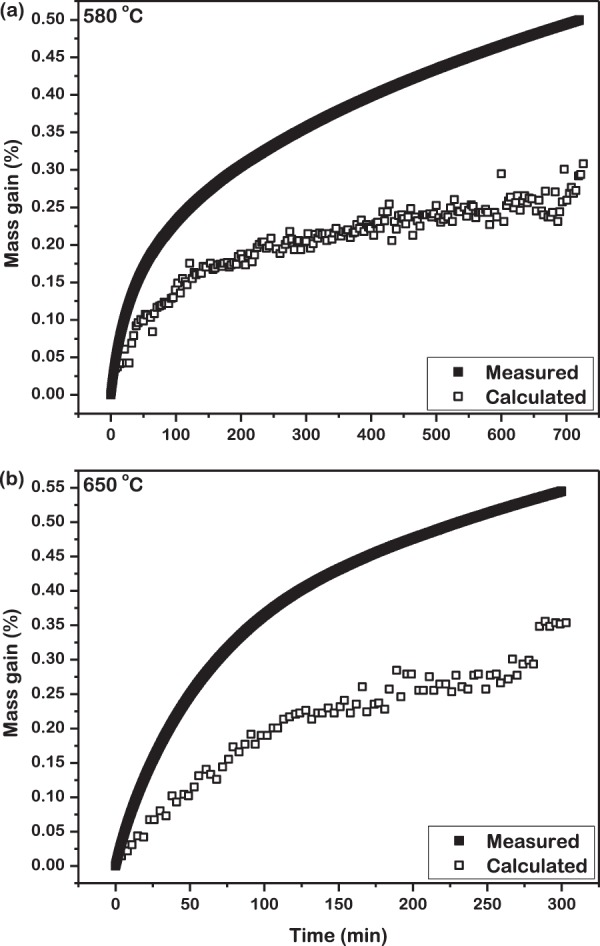
Comparison of the theoretically estimated gain in mass with that measured in practice during isothermal oxidation of powder at (**a**) 580 °C for 720 min and (**b**) 650 °C for 300 min. In practice, non-spherical particles oxidize to yield higher gain in mass than that computed from the scattering model.

In order to examine the accuracy of the quantitative analysis by USAXS, the gain in mass of the powder due to isothermal oxidation, per unit mass of powder was theoretically estimated. Three assumptions were made for this theoretical computation. First, all the particles of this metallic glass powder can be represented by solid spheres of identical diameter equal to 40 μm. Second, oxidation of the particles result in the formation of only one oxide, Fe_2_O_3_. Third, the thickness of oxide formed on each particle is equal. Based on these assumptions, the initial mass of a single powder particle was calculated from its diameter and density (7500 kg m^−3^)^17^. The mass of oxide was calculated from its thickness and density (5240 kg m^−3^). The percentage gain in mass was estimated by dividing the mass of oxide formed on a single particle by the mass of that particle. The results from this theoretical estimation was compared with the gain in mass measured *in*-*situ* during isothermal oxidation by a thermogravimetric analyzer. This is presented in Fig. 8 for the temperatures 580 °C and 650 °C. It is observed that the theoretically estimated gain in mass continuously underestimates that actually measured in practice. This can be explained on the basis of the assumptions and the mechanism of oxidation. The isothermal oxidation investigated here is controlled by diffusion at the surface with the rate being dependent on the surface area of the powder particles. For a given volume, the surface area is least for a sphere. This theoretical calculation performed assuming all particles hence estimates a lower gain in mass. In practice, non-spherical particles oxidize to yield a higher gain in mass that is measured by the thermogravimetric analyzer as observed in Fig. 8.

## Conclusions

Oxidation of Fe_48_Cr_15_Mo_14_Y_2_C_15_B_6_ metallic glass powder at 580 °C upto 720 min and 650 °C upto 300 min results primarily in the formation of polycrystalline Fe_2_O_3_. This oxide grows over the entire surface of the powder particles as a uniform shell which further constitutes of multiple grains. The ultra-small angle X-ray scattering intensity acquired *in*-*situ* during isothermal oxidation and modelled based on this hierarchical structure of the oxide, enumerated the increase in thickness of the oxide with time, rapid initially which gradually decreased. The relative gain in mass due to oxidation computed theoretically from this model, relatively underestimates that measured in practice owing to the distribution in size of the pristine particles. Overall, the results presented here establish the chemical identification, structural analysis and quantitative computation of isothermal oxidation in metallic glass powder. In the broad perspective, this manifests the potential to unfold new paradigms of research on interfacial reactions in powder materials at elevated temperatures and develop predictive analytical capabilities built on complementary *ex*-*situ* and *in*-*operando* scattering techniques.

## Methods

### Metallic glass powder

The composition of the atomized metallic glass powder used in this investigation was Fe_48_Cr_15_Mo_14_Y_2_C_15_B_6_ (at.%). The morphology of the pristine powder particles was observed in a scanning electron microscope (FEI, Quanta 600). Size of individual particles was measured from the secondary electron micrographs using a public domain image processing software, ImageJ (available from the National Institute of Health, USA).

### Differential scanning calorimetry

Thermal behavior of the pristine metallic glass powder was evaluated using a simultaneous thermal analyzer (TA Instruments, SDT Q600) under dry high purity argon gas atmosphere at a flow rate of 50 ml min^−1^. About 100 mg of the powder was heated in an alumina crucible, continuously from ambient temperature to 900 °C, at a constant heating rate of 50 °C min^−1^.

### Isothermal oxidation

*Ex*-*situ* isothermal oxidation of the metallic glass powder was carried out in a muffle furnace (Thermo Fisher Scientific, FB1315M) in freely flowing dry air at atmospheric pressure at 580 °C and 650 °C. For each experiment, the furnace was first preheated to the desired temperature. After the temperature stabilized, about 3 g of the powder was placed in an alumina crucible and introduced inside the furnace. Isothermal time dependent experiments were conducted at the lower temperature, 580 °C for 120; 240; 360; 480; 600 and 720 min and at the higher temperature, 650 °C for 60; 120; 180; 240 and 300 min respectively. Temperature was controlled within ±0.3 °C during all the experiments.

### Single-particle Raman spectroscopy

Chemical identification of the oxides was conducted by Raman spectroscopy. Spectra were acquired by a micro-Raman spectrometer (WITec, alpha300 R) equipped with a grating of 600 lines mm^−1^. A laser wavelength of 532 nm was utilized to excite the spectra with an incident laser power of 3 mW. The laser spot size on the samples was 10 μm in diameter. The signal was accumulated for 200 s by employing a 20X objective lens.

### X-ray diffraction

Structural analysis of the pristine and oxidized metallic glass powder was performed using an X-ray diffractometer (PANalytical, PW 1830) operated with Cu-K*α* radiation (*λ* = 1.5418 Å). At least three measurements were performed for each sample of oxidized powder to ensure reliability of the results and a representative spectra was plotted.

### *In-situ* ultra-small angle X-ray scattering

*In*-*situ* ultra-small angle X-ray scattering experiments were performed during isothermal oxidation of the metallic glass powder. For each experiment, a sample ‘sandwich’ was prepared using mica sheets and the metallic glass powder as described here. First, a circular sheet of mica with thickness 25 μm and diameter 6 mm was laid out horizontally. Another annular sheet of mica of the same thickness with external diameter of 6 mm and internal aperture of 3 mm was placed on top of and concentric to the first sheet. A thin layer of the metallic glass powder was spread uniformly within the internal aperture of the annular mica sheet. Thus, the annular sheet behaved as a ‘washer’, thereby securing the powder sample in place. A third sheet of mica, identical to the first one, was placed on top, covering the powder and the annular sheet. A thin sample with the metallic glass powder sandwiched between mica sheets was thus assembled. In this configuration air flowed freely at atmospheric pressure. This assembly was introduced within the sample cell of a temperature controlled stage (Linkam Scientific, TS1500) with a temperature stability of 1 °C. The temperature of the sample cell in the stage was increased from ambient to the desired values of 580 °C and 650 °C at a heating rate of 50 °C min^−1^. Isothermal time dependent experiments were conducted at the lower temperature, 580 °C for 720 min and at the higher temperature, 650 °C for 300 min.

X-ray scattering intensities were measured as a function of the scattering vector in the range of 0.0005 to 0.2 Å^−1^, *in*-*situ* during isothermal oxidation, by the Ultra-Small Angle X-ray Scattering instrument at beamline 9-ID of the Advanced Photon Source, Argonne National Laboratory^48,49,74^. The instrument was operated with an X-ray wavelength of 0.59 Å that corresponds to an X-ray energy of 21 keV, beam size of 0.8 × 0.8 mm and X-ray photon flux of approximately 10^13^ mm^−2^ s^−1^. The exposure time for each measurement was 1 min and the duration between two consecutive measurements was approximately 3 min. Background scattering from the environment, instrument and temperature controlled stage was subtracted from the measured intensities by the USAXS data reduction package, INDRA. The small angle scattering data analysis tool suite IRENA was utilized for modeling the reduced scattering intensities. X-ray scattering length densities and the corresponding X-ray scattering contrasts of the materials were calculated based on their compositions by the scattering contrast calculator support tool, also available in IRENA^56^.

### Thermogravimetric analysis

The gain in mass was measured *in*-*situ* during isothermal oxidation of the metallic glass powder at 580 °C and 650 °C by a simultaneous thermal analyzer (TA Instruments, SDT Q600). About 100 mg of the powder was placed in an alumina crucible and the temperature of the sample was increased from ambient to the desired values of 580 °C and 650 °C at a heating rate of 50 °C min^−1^. The gain in mass was measured continuously during isothermal time dependent experiments at the lower temperature, 580 °C for 720 min and at the higher temperature, 650 °C for 300 min.

## Supplementary information

LaTeX Supplementary File

Supplementary Material

## Data Availability

The data that support the findings of this study are available from the corresponding author upon reasonable request.
