# Supplementary figures and images for: Quantification of Thermal Oxidation in Metallic Glass Powder using Ultra-small Angle X-ray Scattering
Source: Sci Rep. 2019 May 2;9:6836. doi: 10.1038/s41598-019-43317-0 (PMC6497630; doi:10.1038/s41598-019-43317-0)

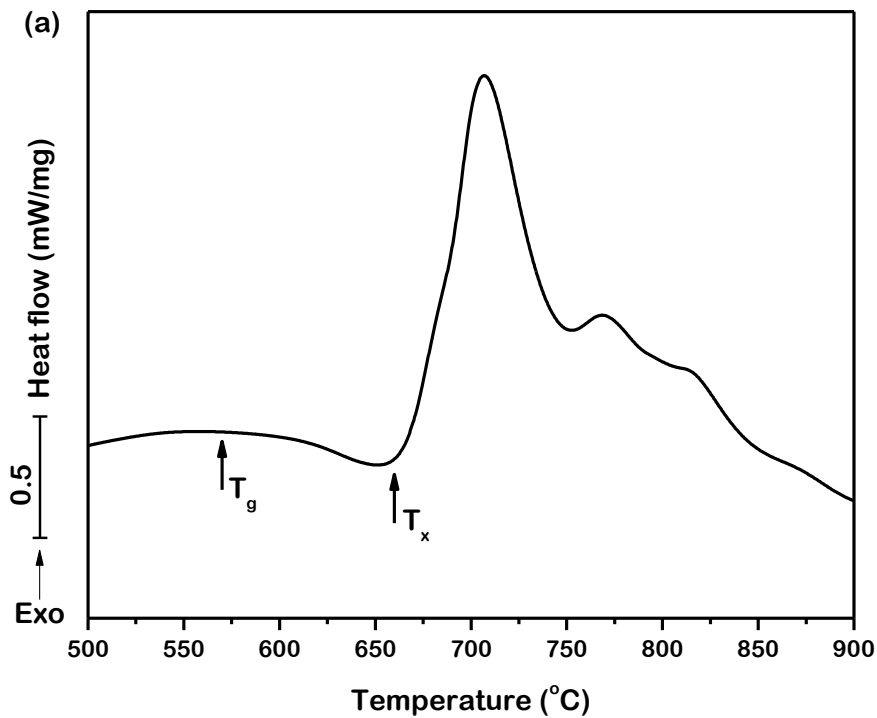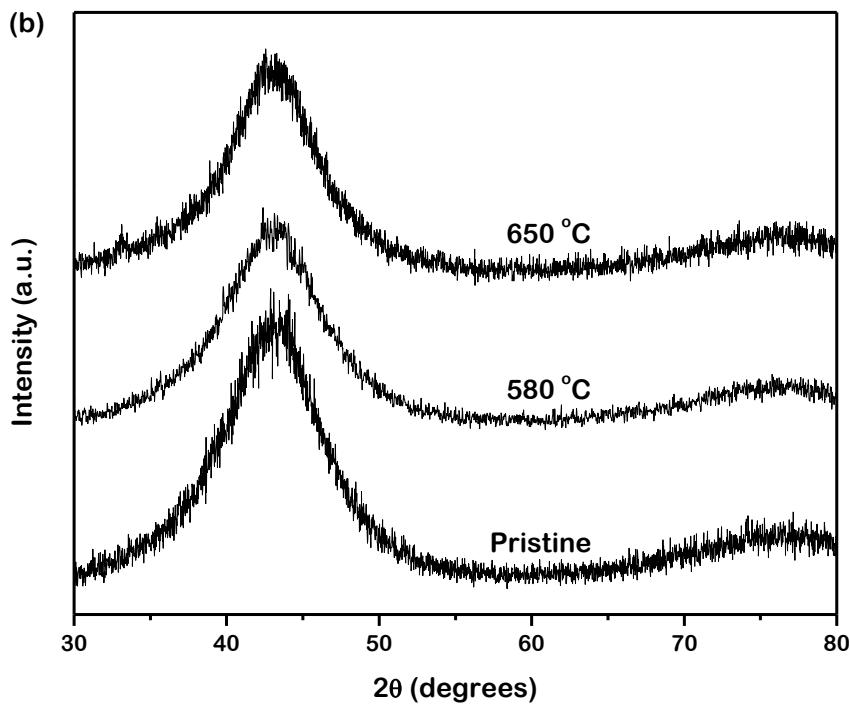

Supplement: Supplementary file 1 — LaTeX Supplementary File [file 41598_2019_43317_MOESM1_ESM.zip › dscxrd.pdf]

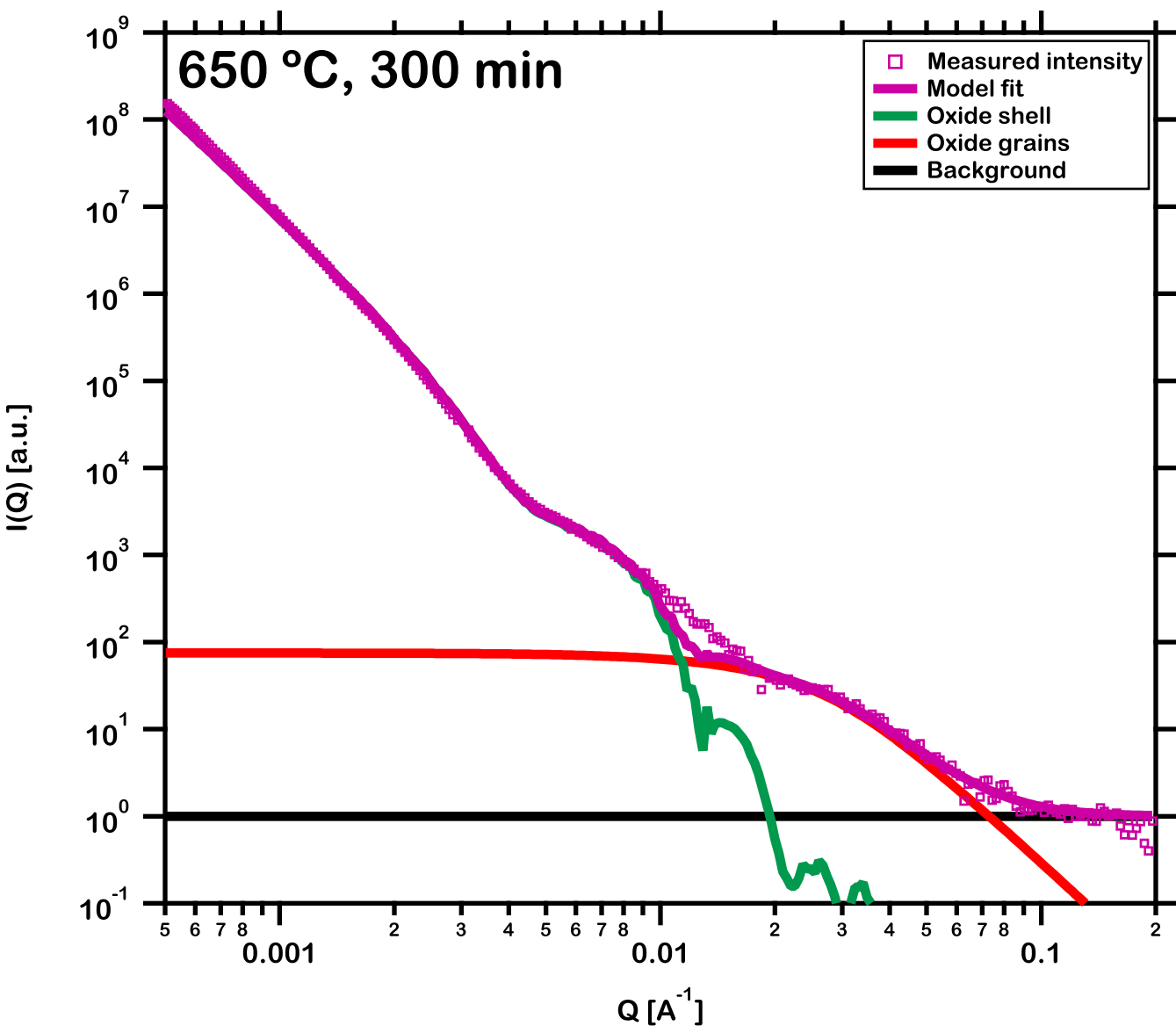

Supplement: Supplementary file 1 — LaTeX Supplementary File [file 41598_2019_43317_MOESM1_ESM.zip › fit.pdf]

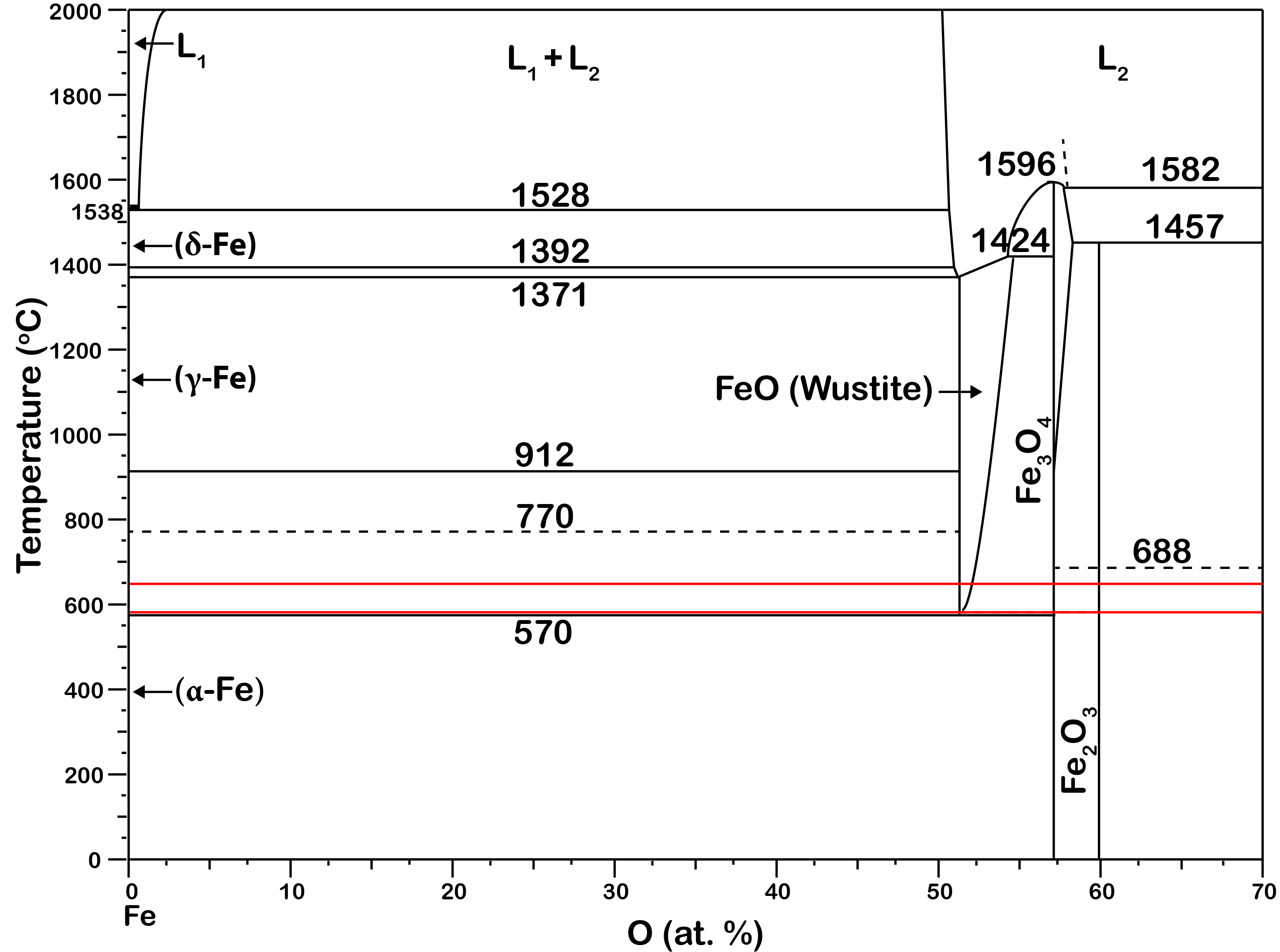

Supplement: Supplementary file 1 — LaTeX Supplementary File [file 41598_2019_43317_MOESM1_ESM.zip › phasediagram.jpg]

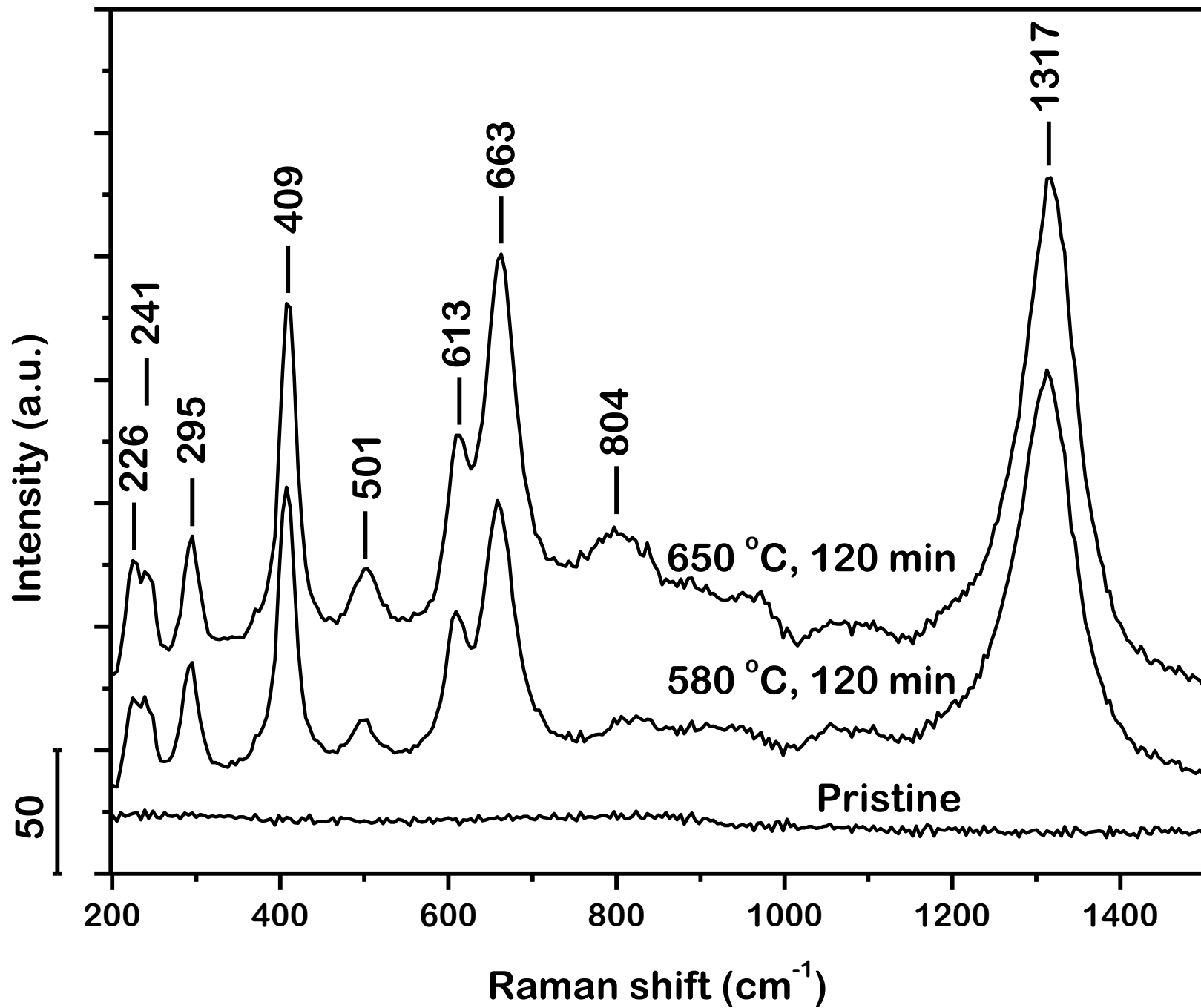

Supplement: Supplementary file 1 — LaTeX Supplementary File [file 41598_2019_43317_MOESM1_ESM.zip › raman1.pdf]

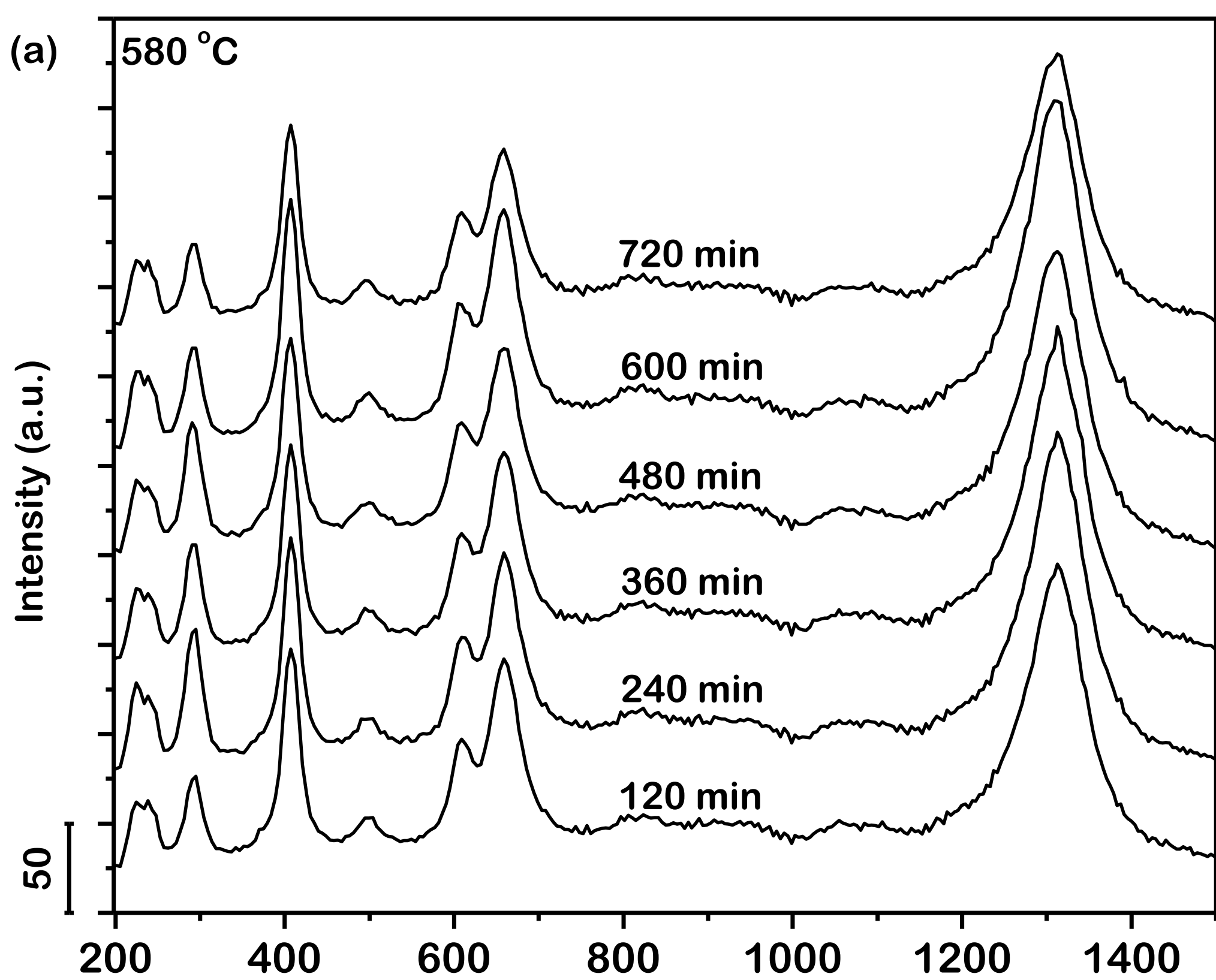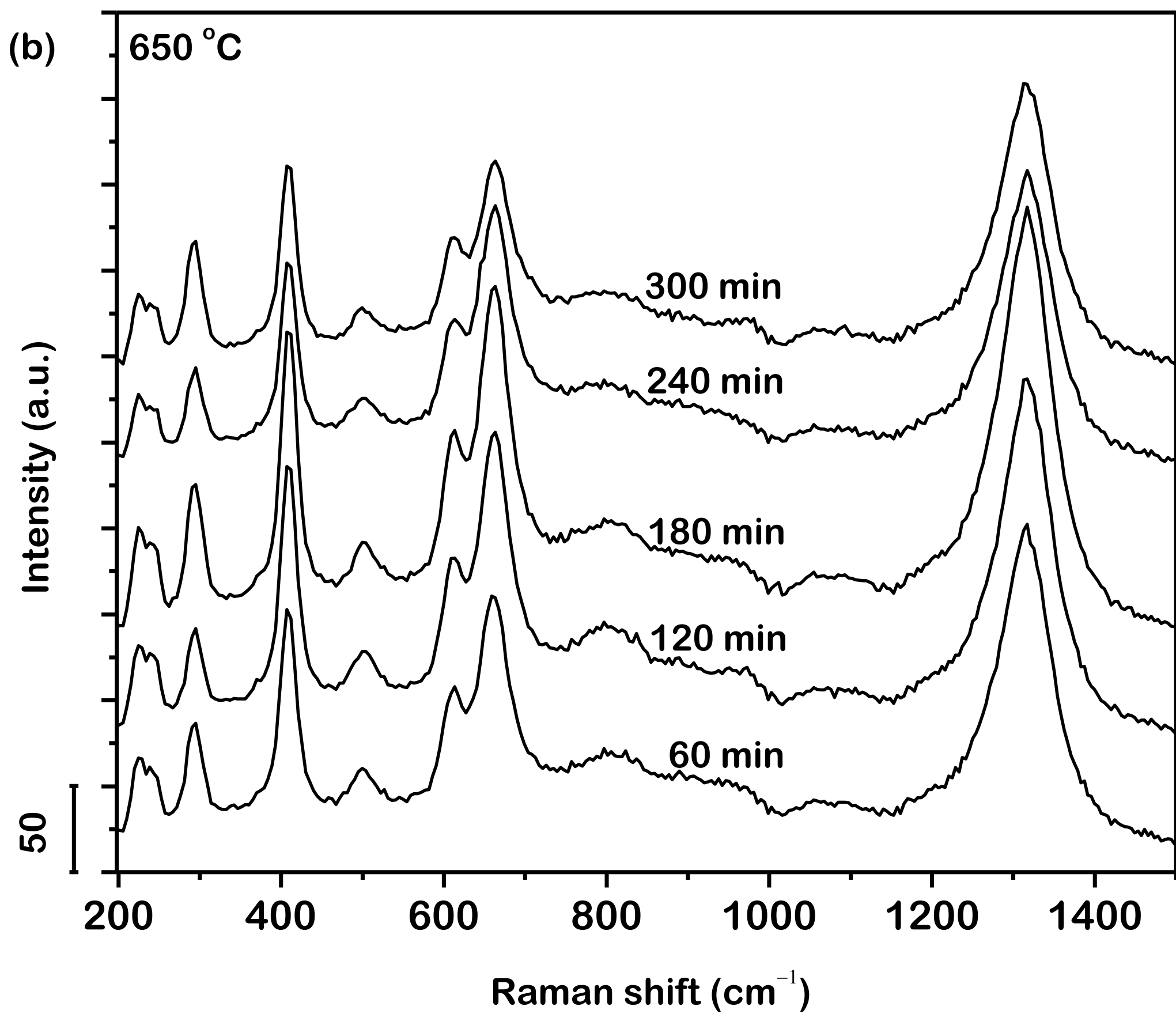

Supplement: Supplementary file 1 — LaTeX Supplementary File [file 41598_2019_43317_MOESM1_ESM.zip › raman2.pdf]

(a)

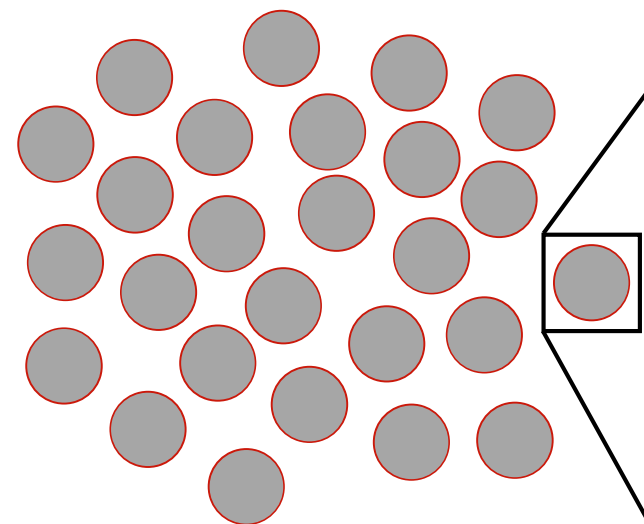

100  $\mu\text{m}$

(b)

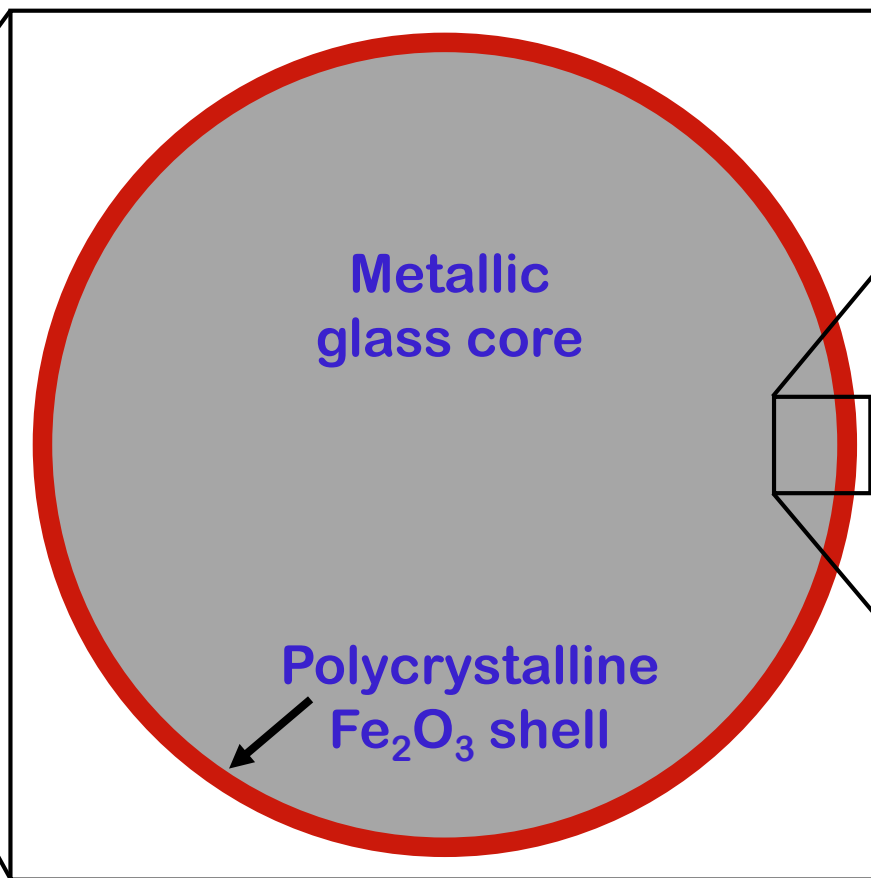

10  $\mu\text{m}$

(c)

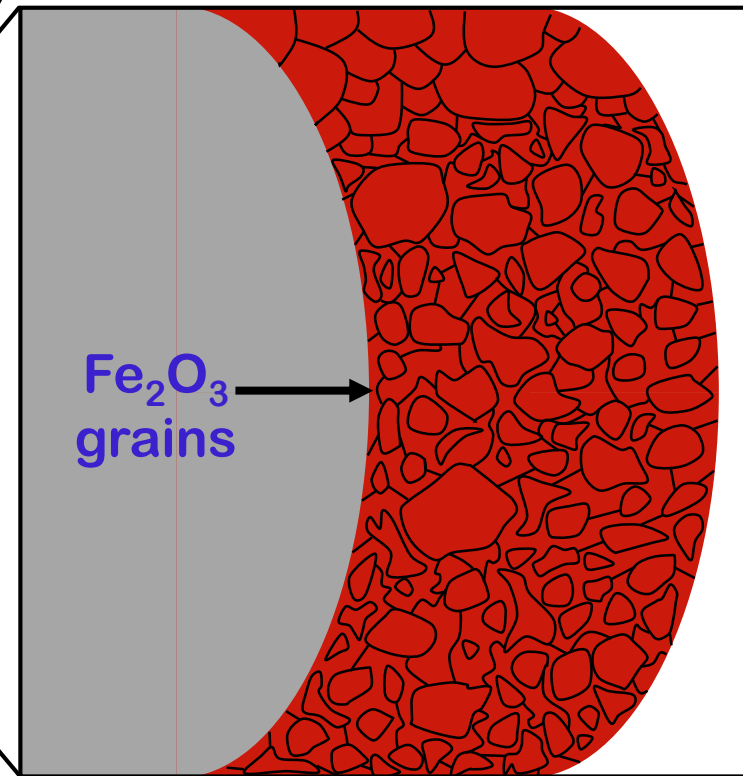

50 nm

Supplement: Supplementary file 1 — LaTeX Supplementary File [file 41598_2019_43317_MOESM1_ESM.zip › schematic.pdf]

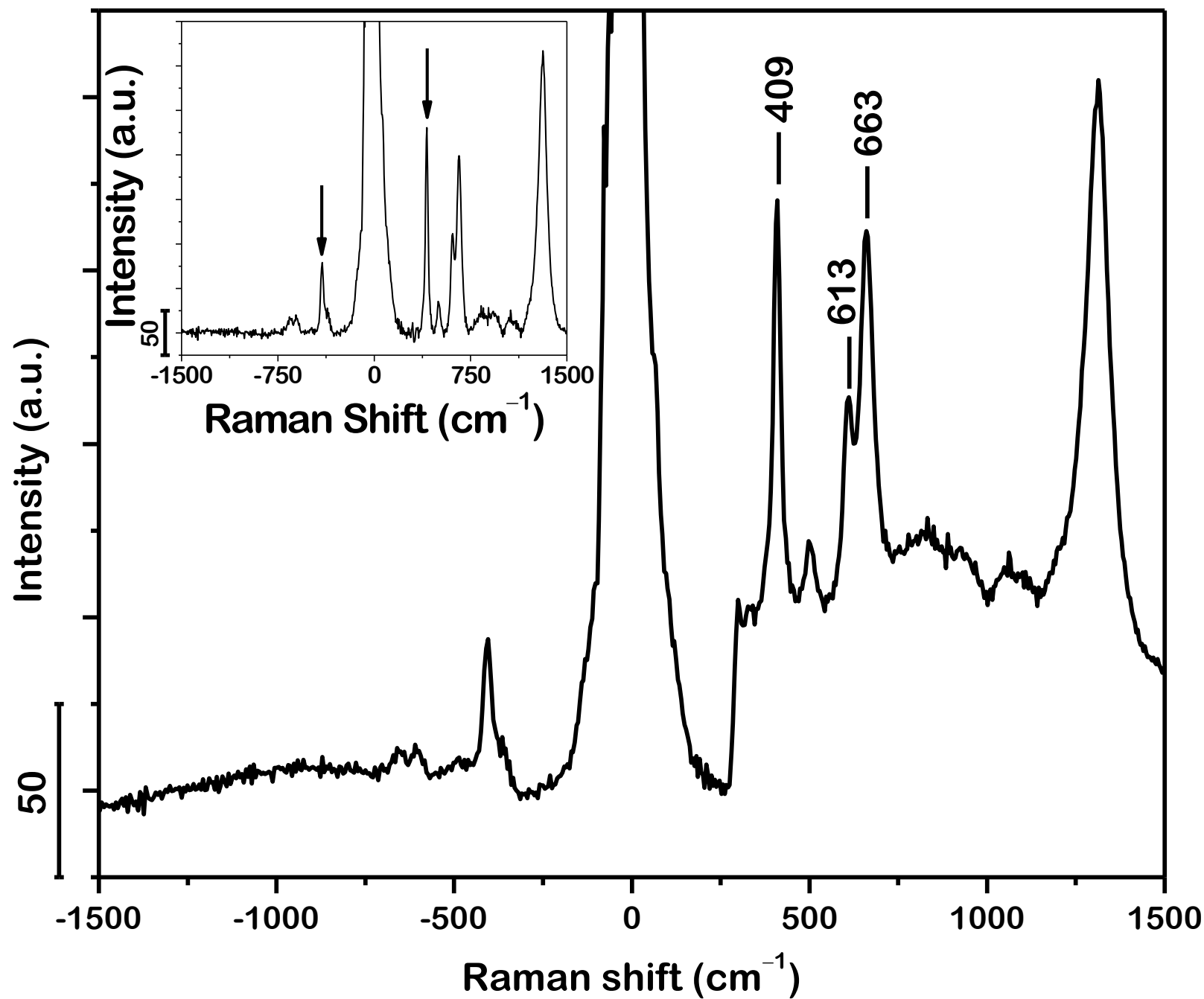

Supplement: Supplementary file 1 — LaTeX Supplementary File [file 41598_2019_43317_MOESM1_ESM.zip › stokes.pdf]

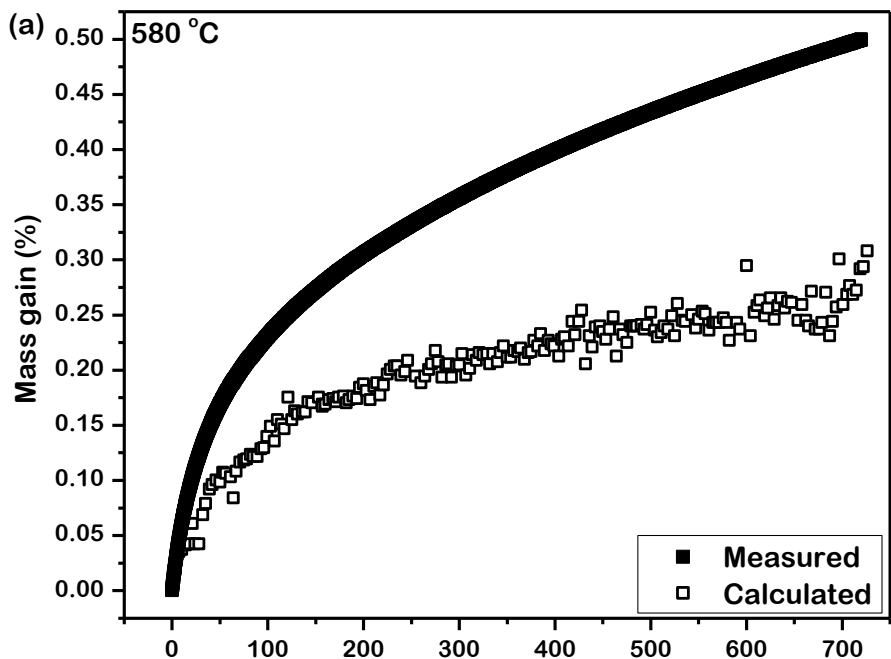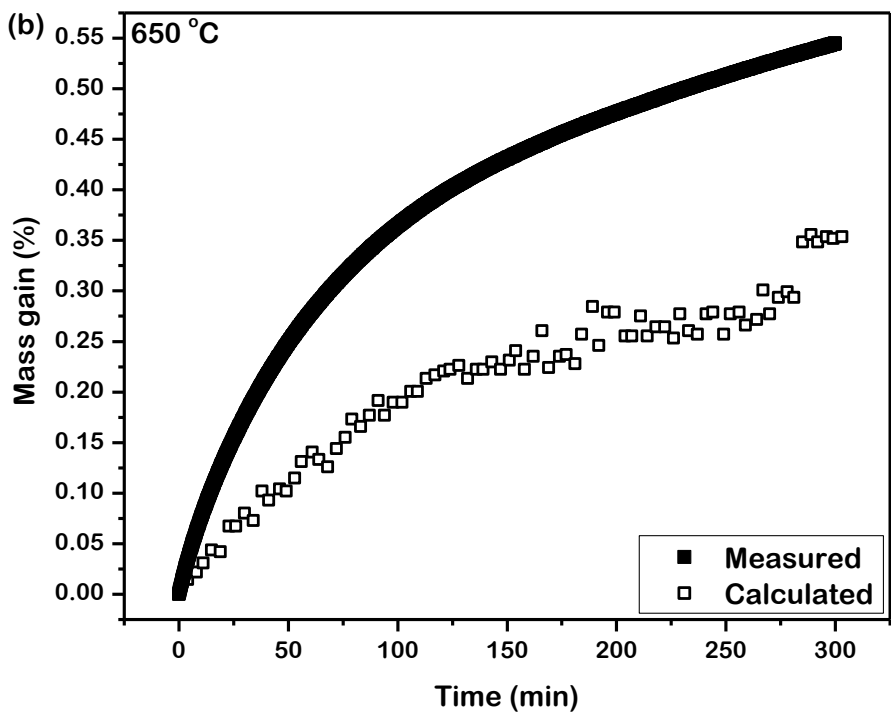

Supplement: Supplementary file 1 — LaTeX Supplementary File [file 41598_2019_43317_MOESM1_ESM.zip › tga.pdf]

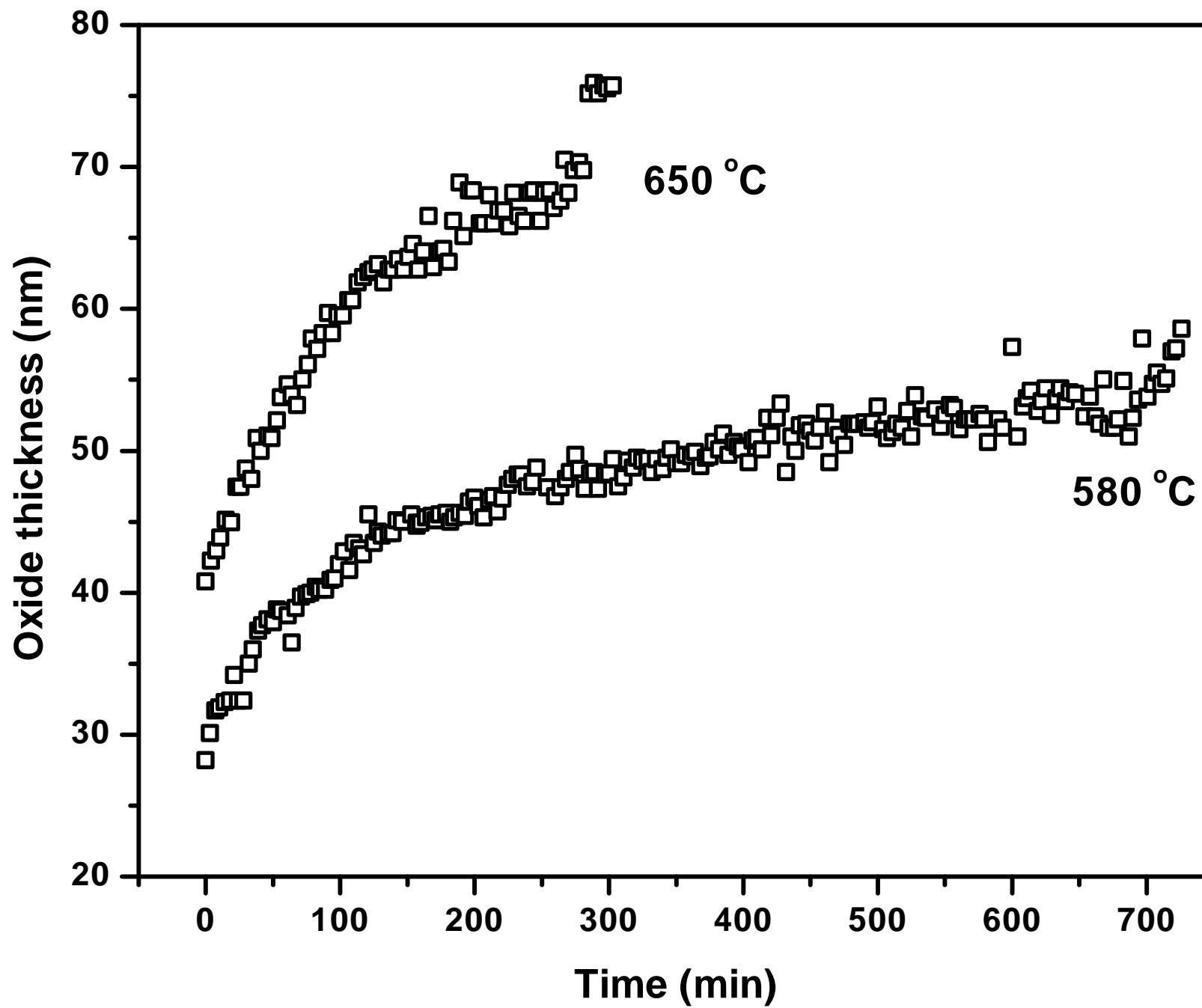

Supplement: Supplementary file 1 — LaTeX Supplementary File [file 41598_2019_43317_MOESM1_ESM.zip › thickness.pdf]

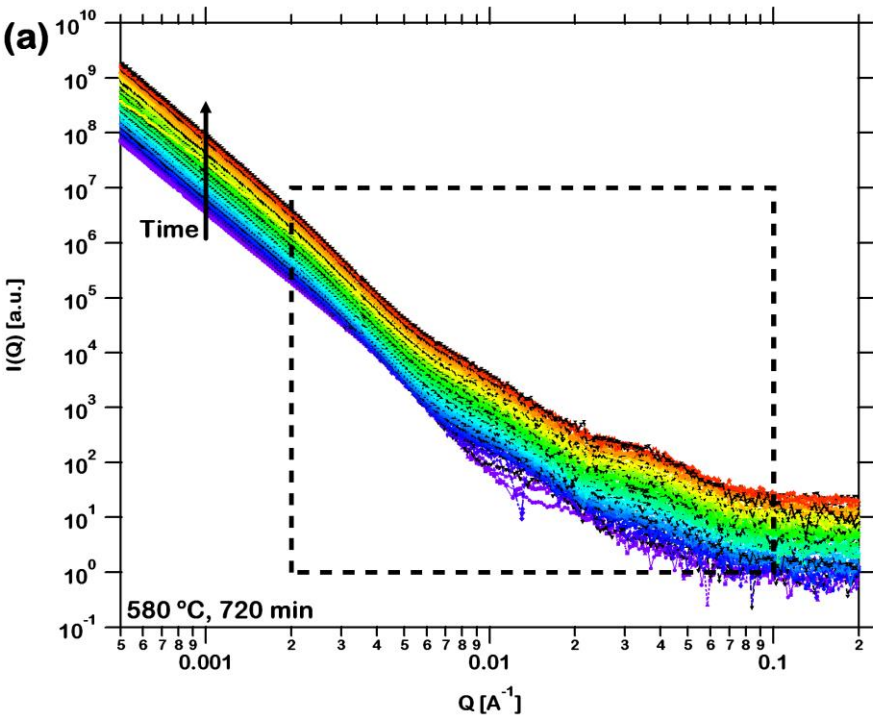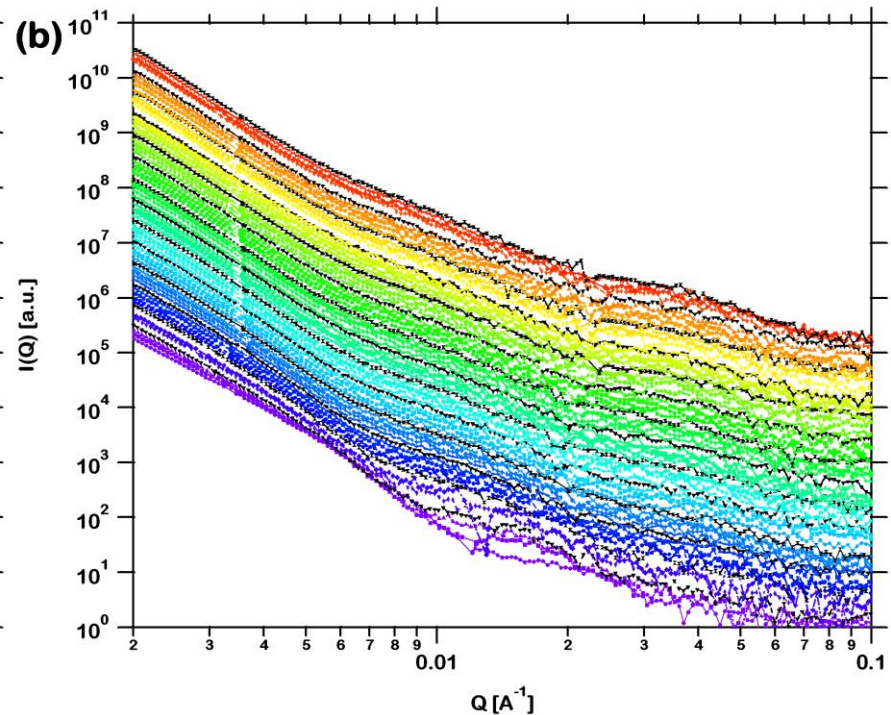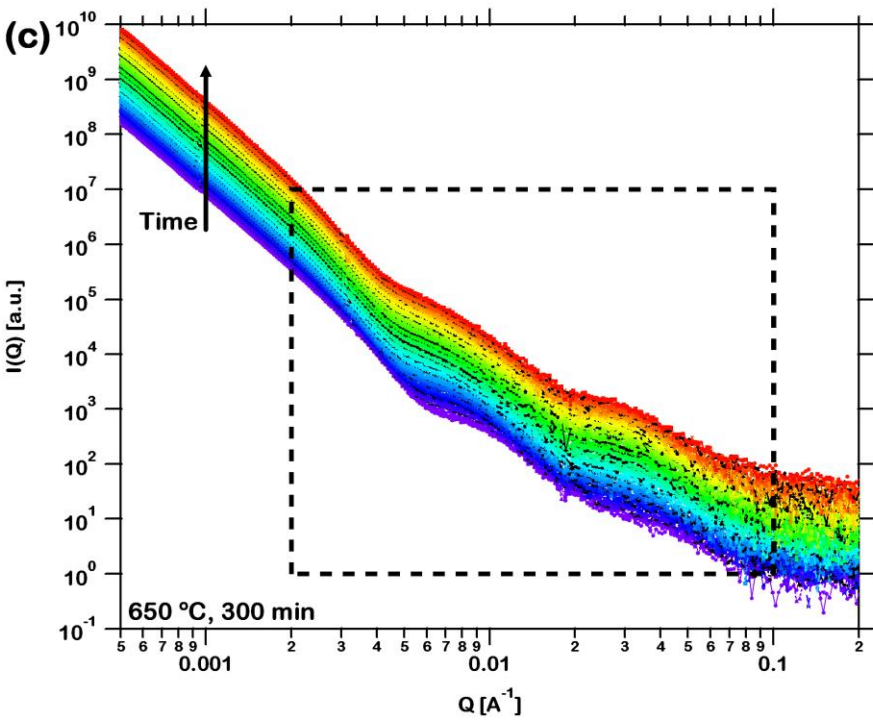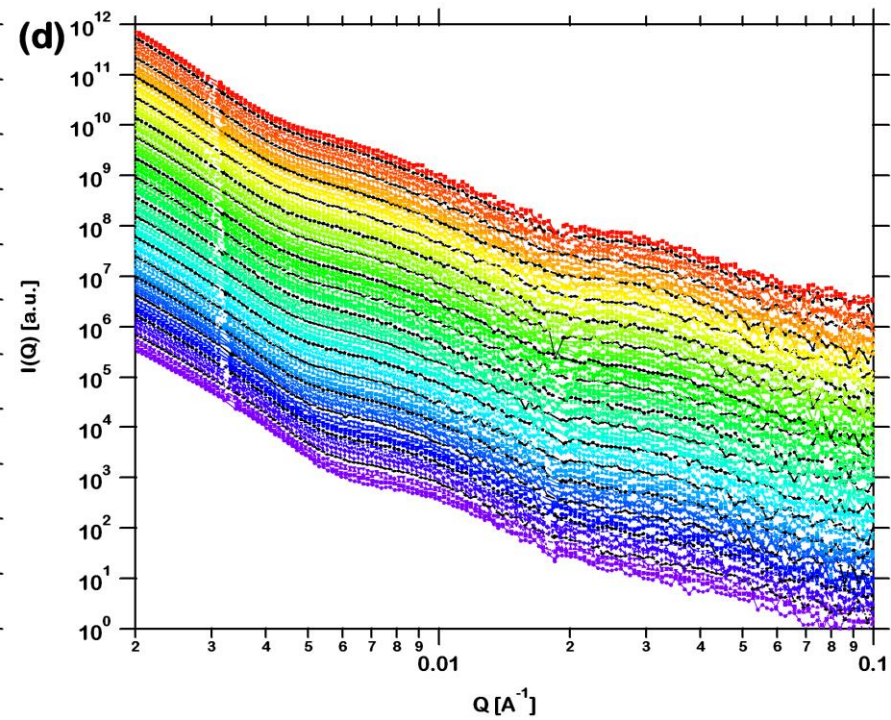

Supplement: Supplementary file 1 — LaTeX Supplementary File [file 41598_2019_43317_MOESM1_ESM.zip › usaxsoxdn.pdf]

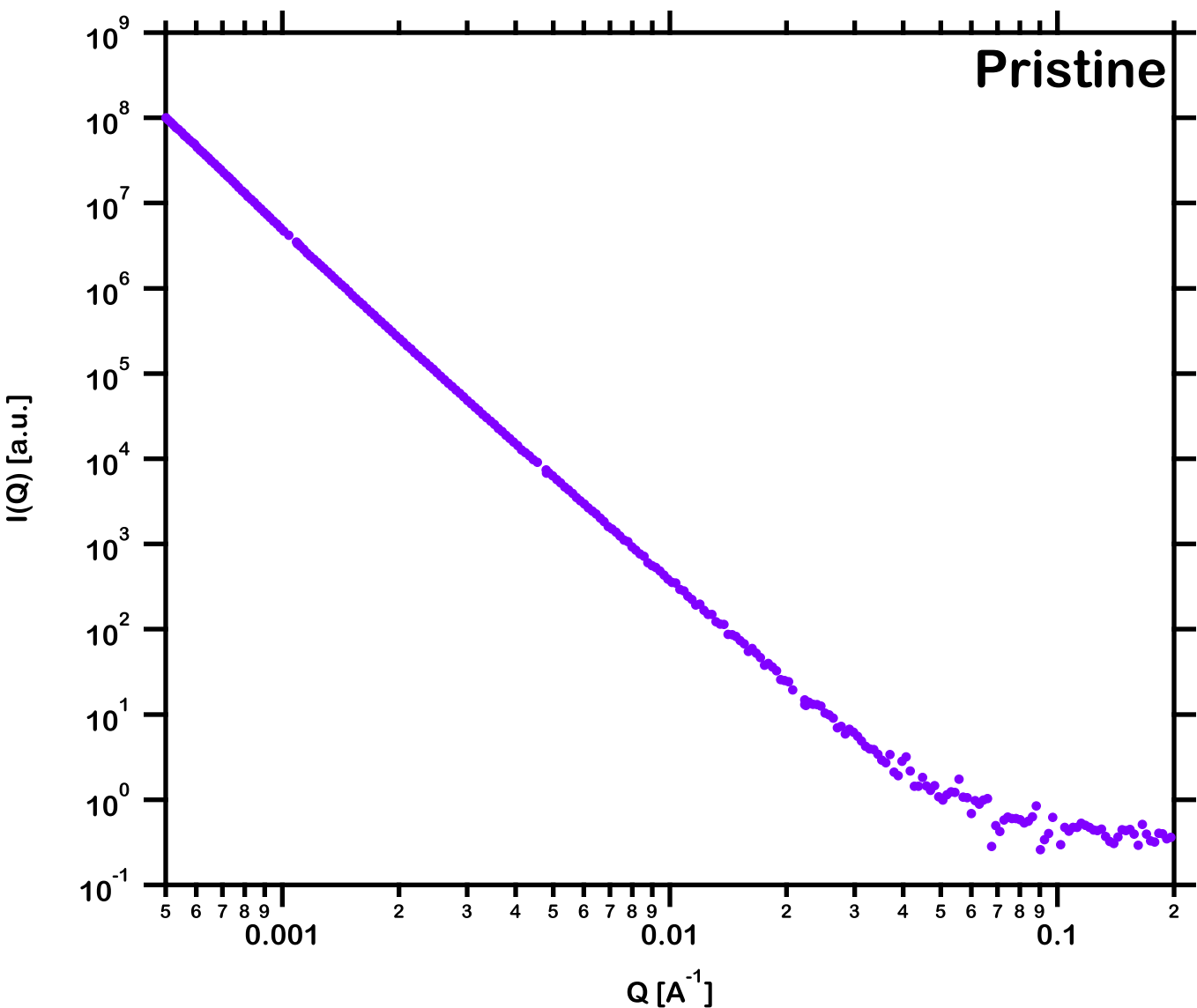

Supplement: Supplementary file 1 — LaTeX Supplementary File [file 41598_2019_43317_MOESM1_ESM.zip › usaxsroom.pdf]

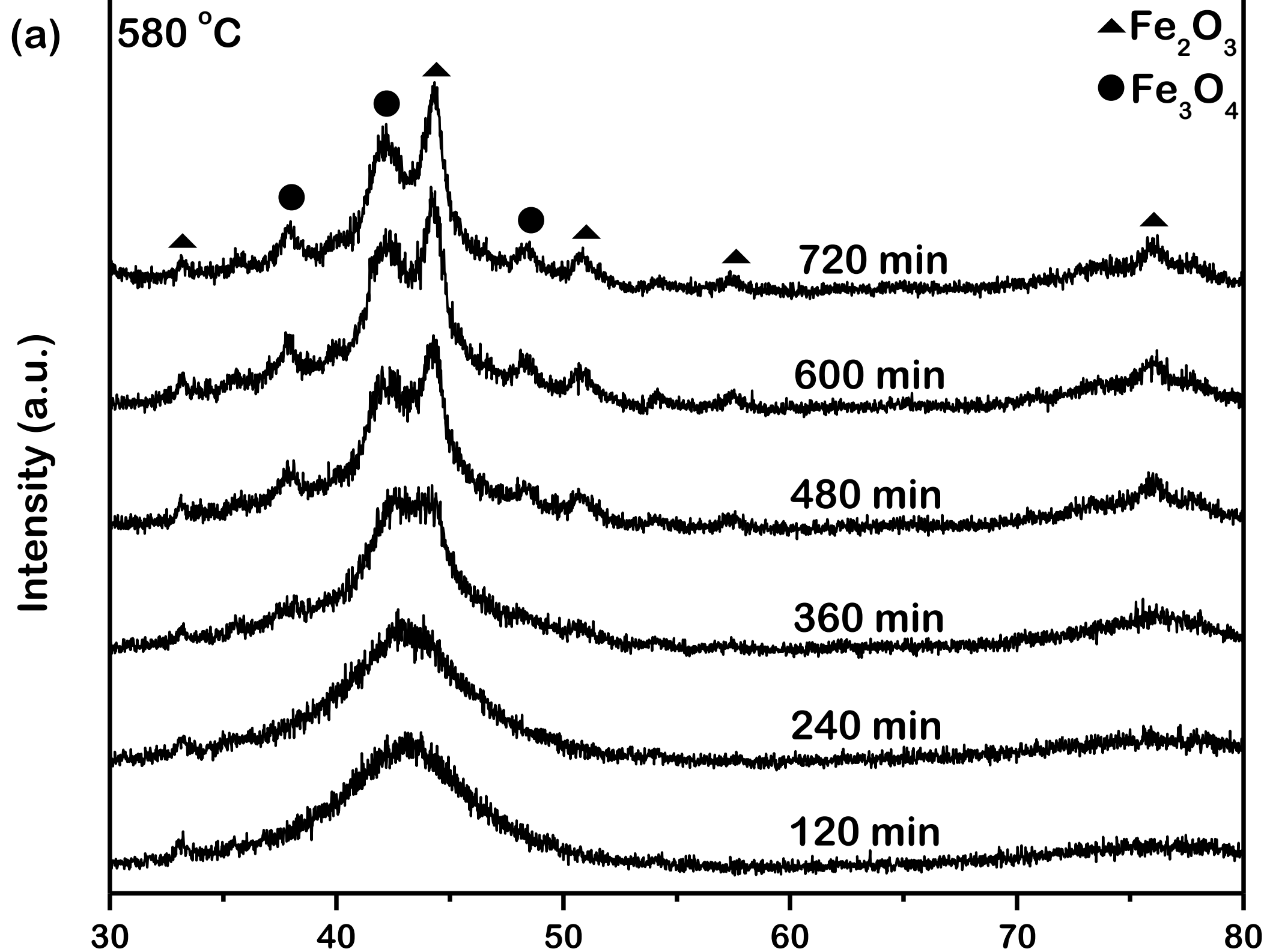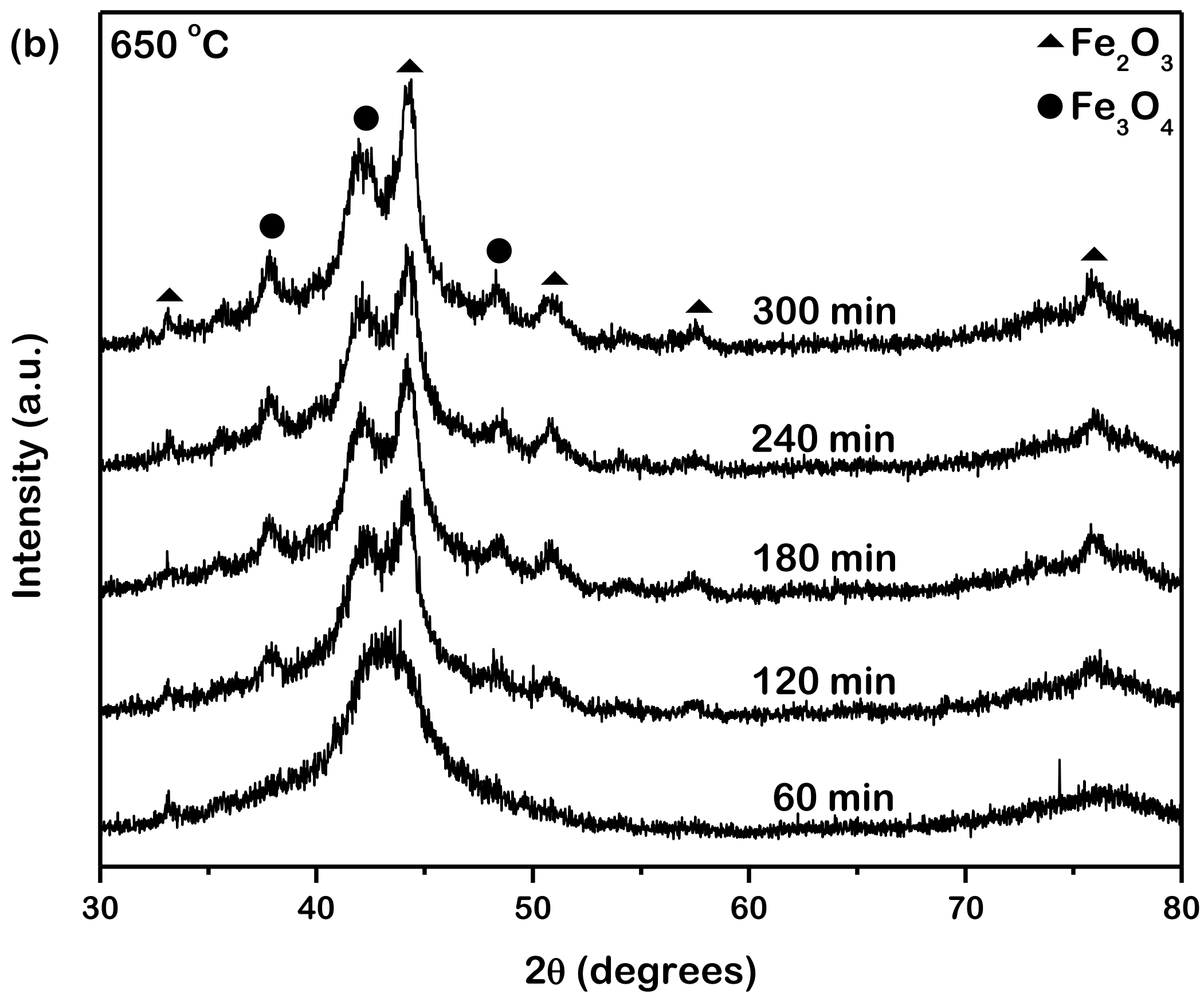

Supplement: Supplementary file 1 — LaTeX Supplementary File [file 41598_2019_43317_MOESM1_ESM.zip › xrd.pdf]
