# Supplementary material for: Quantification of Thermal Oxidation in Metallic Glass Powder using Ultra-small Angle X-ray Scattering
Source: Sci Rep. 2019 May 2;9:6836. doi: 10.1038/s41598-019-43317-0 (PMC6497630; doi:10.1038/s41598-019-43317-0)
Supplement: Supplementary file 2 — Supplementary Material [file 41598_2019_43317_MOESM2_ESM.pdf]

Supplementary Material for

Quantification of Thermal Oxidation in Metallic Glass Powder using Ultra-small Angle X-ray Scattering

Tanaji Paul[1,3], Linqi Zhang[1], Sourabh Biswas[1], Archana Loganathan[2], Matthew G. Frith[3], Jan Ilavsky[3], Ivan Kuzmenko[3], Jim Puckette[4], A. Kaan Kalkan[1], Arvind Agarwal[2] and Sandip P. Harimkar[1,*]

[1]School of Mechanical and Aerospace Engineering, Oklahoma State University, Stillwater, OK 74078, United States

[2]Plasma Forming Laboratory, Department of Mechanical and Materials Engineering, Florida International University, Miami, FL 33174, United States

[3]X-ray Science Division, Advanced Photon Source, Argonne National Laboratory, 9700 South Cass Avenue, Argonne, IL 60439, United States

[4]Boone Pickens School of Geology, Oklahoma State University, Stillwater, OK 74078, United States

*Corresponding Author: sandip.harimkar@okstate.edu

Powder morphology and structure

The morphology of the pristine $Fe_{48}Cr_{15}Mo_{14}Y_2C_{15}B_6$ metallic glass powder consists of particles of various shapes with the majority of them being spherical. The sizes of the particles were measured from several SEM micrographs, a representative one of which is presented in Fig. S1.

The isochronal DSC trace of the metallic glass powder, measured at a heating rate of 50 $^oCmin^{-1}$, as presented in Fig. S2(a), exhibited a glass transition temperature T_g of about 570 ^oC and

a crystallization onset temperature T_x 660 ^oC. Based on this thermogram, the temperatures of isothermal oxidation were determined to be 580 ^oC and 650 ^oC. The XRD spectra acquired from the powder in both pristine and annealed conditions, presented in Fig. S2(b), exhibited absence of crystallization as confirmed by the diffused peak in each spectra, characteristic of fully amorphous materials.

Iron-oxygen phase diagram

The iron-rich section of the iron-oxygen phase diagram[1] shows that at the temperatures of isothermal oxidation experiments employed in this investigation, 580 ^oC and 650 ^oC, represented by red lines, the oxides that are thermodynamically stable are FeO, Fe_2O_3 and Fe_3O_4.

USAXS intensity acquired from pristine powder

The USAXS intensity, I(Q) acquired from this pristine metallic glass powder at ambient temperature over a scattering vector, Q-range of 0.0005 A^{-1} to 0.2 A^{-1}, in accord with the expected Q-dependence for a dilute solution of identical uniform spheres at $Q >> D^{-1}$, can be observed to decay following a power-law as Q^{-4}. This confirms that the powder consists of particles with a smooth surface devoid of any structural features.

Oxide shell thickness

The fitting routine is employed to the USAXS intensity distributions to estimate the evolution of oxide shell thickness during the isothermal experiments. The increase in the oxide shell thickness is rapid at the initial stage and slows down at the later stage as presented in Fig. S5. This thickness is utilized to estimate the mass gain that is compared with that measured by thermogravimetric analysis.

Supplementary Figures

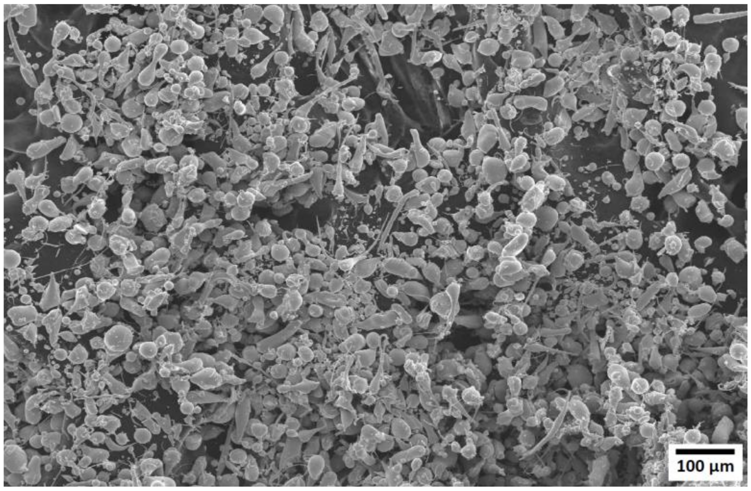

Figure S1. Representative SEM micrograph showing morphology of pristine $Fe_{48}Cr_{15}Mo_{14}Y_2C_{15}B_6$ metallic glass powder

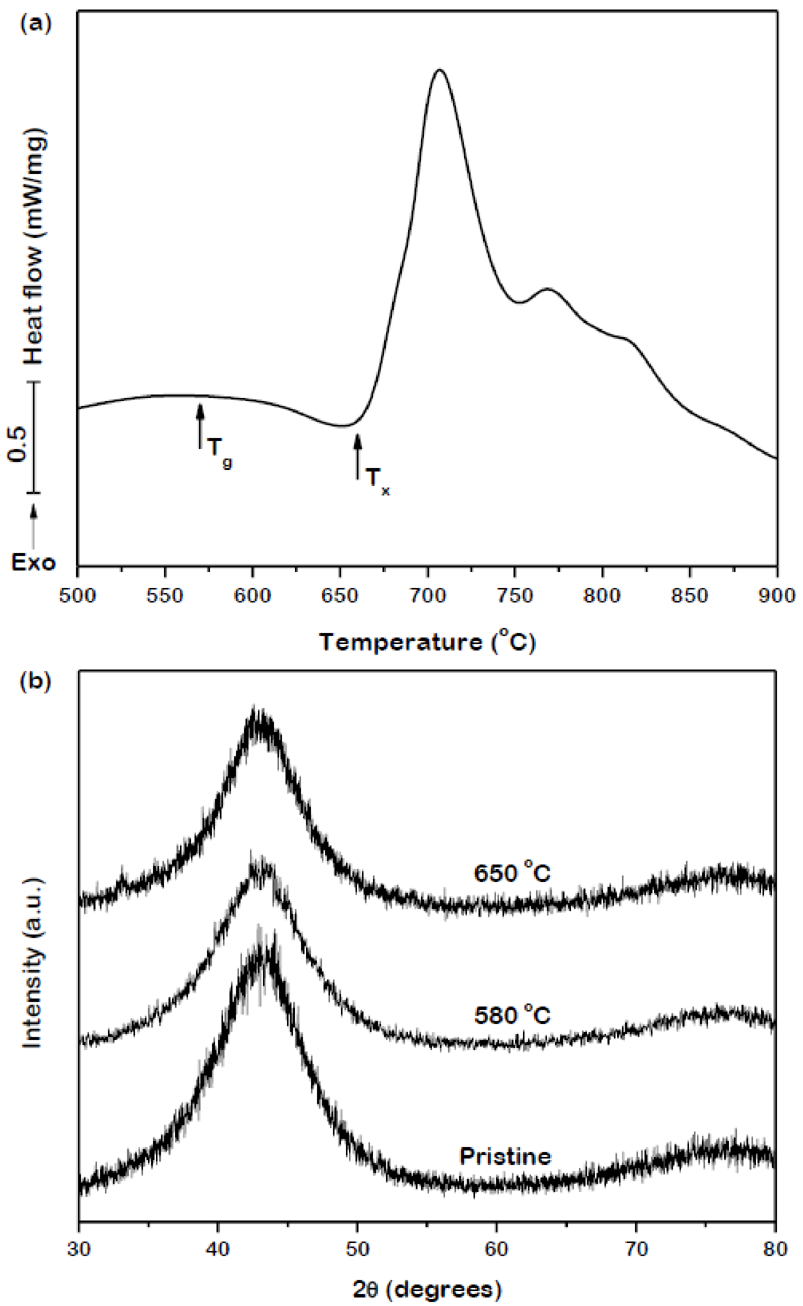

Figure S2. (a) Isochronal DSC trace of the metallic glass powder, measured at a heating rate of 50 $^{o}Cmin^{-1}$ and (b) XRD spectra acquired from the powder in pristine and annealed conditions

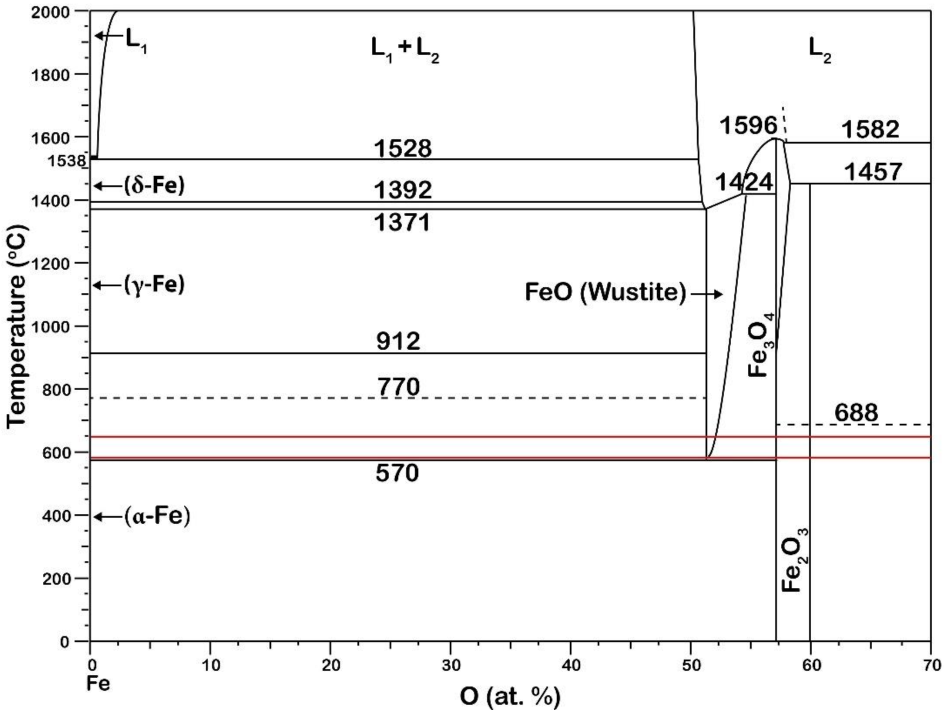

Figure S3. Iron-rich section of the iron-oxygen phase diagram adapted from [1]. Temperatures of isothermal oxidation experiments employed in this investigation, 580 °C and 650 °C, are represented by red lines

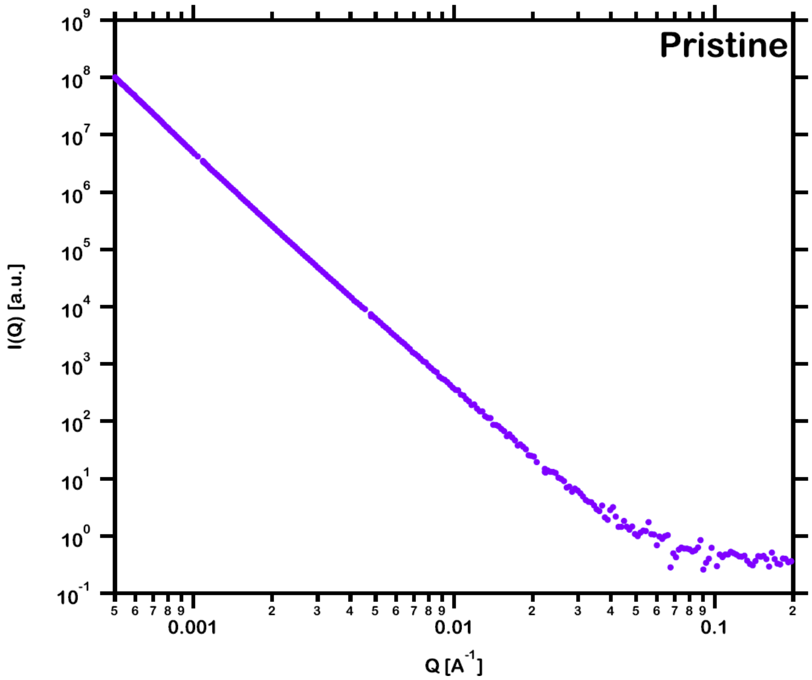

Figure S4. USAXS intensity acquired from pristine metallic glass powder at ambient temperature

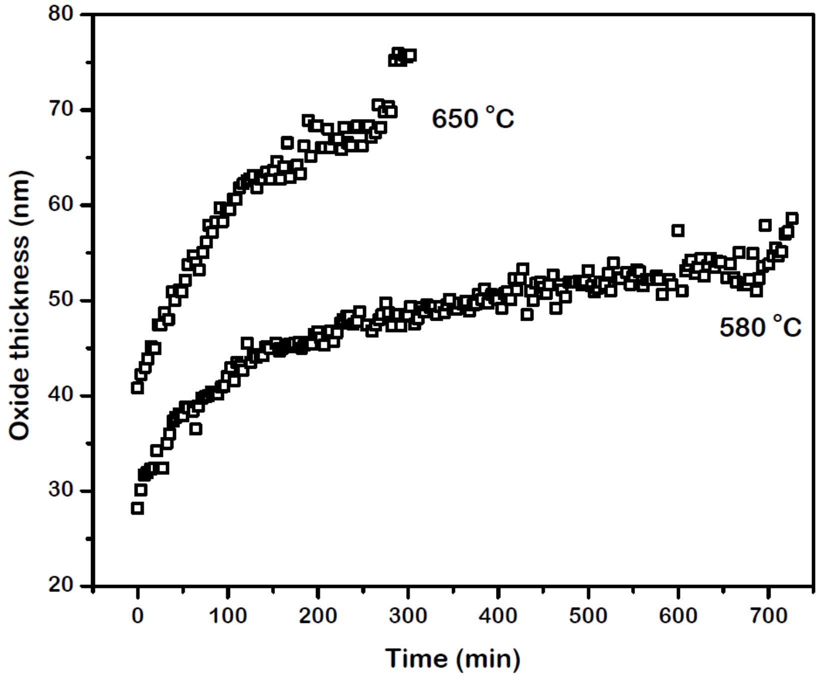

Figure S5. Evolution of oxide shell thickness during isothermal experiments estimated from fitting the USAXS intensity distributions

References

1. Wriedt. H. The Fe-O (iron-oxygen) system. J. Phase Equilibria 12, 170-200 (1991)
